# Supplementary material for: Quantitative localisation of titanium in the framework of titanium silicalite-1 using anomalous X-ray powder diffraction
Source: Nat Commun. 2024 Sep 5;15:7757. doi: 10.1038/s41467-024-51788-7 (PMC11377426; doi:10.1038/s41467-024-51788-7)
Supplement: Supplementary file 1 — Supplementary Information for 'Quantitative locating titanium in the framework of titanium silicalite-1 by exploiting anomalous X-ray powder diffraction' [file 41467_2024_51788_MOESM1_ESM.pdf]

SUPPLEMENTARY INFORMATION

for

Quantitative locating titanium in the framework of titanium silicalite-1 by exploiting anomalous X-ray powder diffraction

Przemyslaw Rzepka,[1,2,3]† Matteo Signorile,[4]† Thomas Huthwelker,[5] Stefano Checchia,[6] Francesca Rosso,[4] Silvia Bordiga,[4]* Jeroen A. van Bokhoven[2,3]*

[1] *J. Heyrovsky Institute of Physical Chemistry Dolejškova 2155/3, 182 23 Prague 8, Czech Republic*

[2] *Institute for Chemical and Bioengineering, ETH Zurich; 8093 Zurich, Switzerland*

[3] *Paul Scherrer Institute, Center for Energy and Environmental Sciences, PSI; 5232 Villigen, Switzerland*

[4] *Department of Chemistry, NIS and INSTM Reference Centre, Università di Torino, Via G. Quarello 15, I-10135 and Via P. Giuria 7, I-10125, Torino, Italy*

[5] *Swiss Light Source, PSI; 5232 Villigen, Switzerland*

[6] *ID 15A, European Synchrotron Radiation Facility 71 Avenue des Martyrs, 38000 Grenoble, France*

*Corresponding authors. Email: jeroen.vanbokhoven@chem.ethz.ch, silvia.bordiga@unito.it

†These authors contributed equally to this work

Table of Contents

Basic characterization of samples (DR-UV-Vis , FT-IR, Raman)
Crystallographic data (Structural Data for refined models TS-1A and TS-1B)
Pair Distribution Function
DFT calculation data (Structural Data for DFT optimized TS-1 model)
SEM images
Structure of possible Ti-peroxo species

Basic characterization of samples

TS-1A and TS-1B are industrial catalysts provided by Evonik AG. Both materials present diffraction signals typical of the only **MFI** phase and feature a BET specific surface area of 430 and 496 m^2g^{-1} and an overall Ti content (by ICP analysis, expressed as wt% TiO_2) of 2.44 and 2.89, respectively. Diffuse reflectance (DR)-UV-Vis (collected on a Varian Cary 5000 spectrometer), ATR-IR (collected on a Bruker Alpha FT-IR spectrometer, equipped with a diamond ATR element) and Raman (collected on a Bruker RFS100 FT-Raman spectrometer, excitation wavelength 1064 nm) spectra of both samples are shown and compared in Supplementary Figure 1.

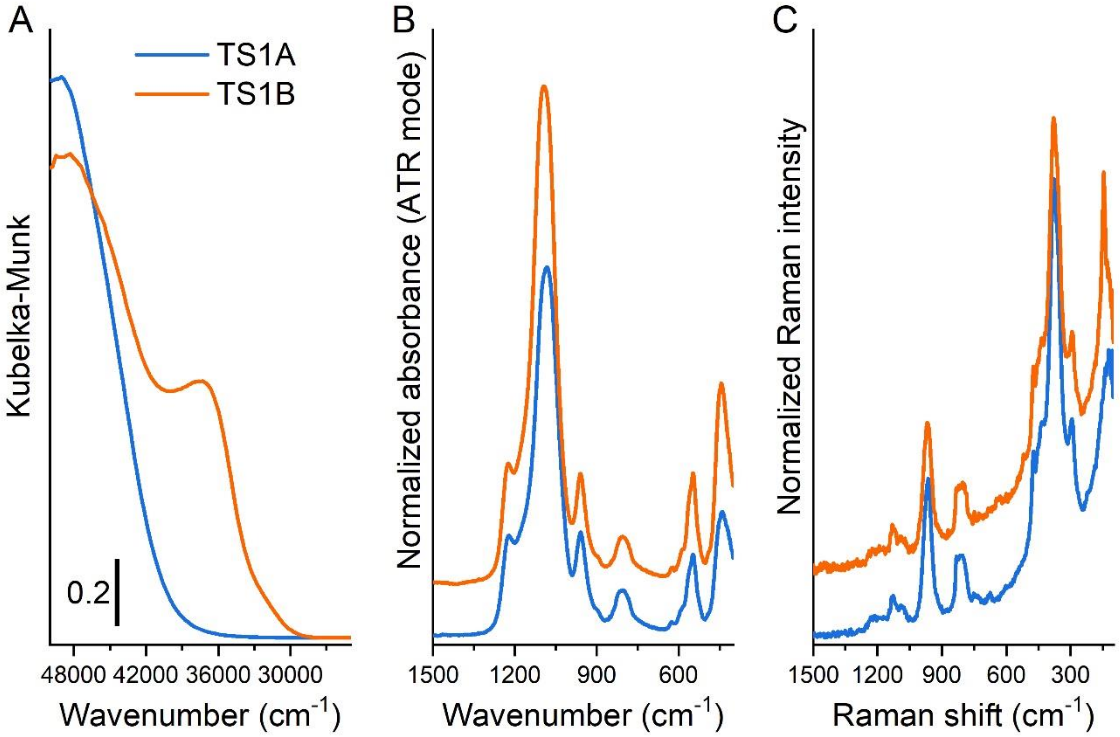

Supplementary Figure 1. DR-UV-Vis (A), ATR-IR (B) and Raman (C) spectra of TS-1A and TS-1B. Data from ref.[1] Intensities of ATR-IR and Raman spectra are normalized to that of the Si-O-Si bridges symmetric stretching mode (800 cm^{-1}), for the sake of quantitative comparison.

DR-UV-Vis data of TS-1A (Figure S1A) shows as the sample features, within the sensitivity of the technique, a single type of Ti sites, namely perfectly tetrahedral

coordinated Ti isomorphously substituting Si in the **MFI** framework (electronic transition peaking at ca. 50000 cm^{-1}). TS-1B present an additional feature at 37000 cm^{-1}, assigned in the literature to different type of Ti species with higher coordination/nuclearity.[2–4] Signorile et al. rigorously demonstrated this electronic transition can be assigned to hexacoordinated (i.e. distorted octahedral) Ti sites, embed in the **MFI** framework as defective positions (missing neighbour Si atoms) and charge-balanced by extraframework cations.[5] Possibly, a small fraction of bulk TiO_2 is also present, as indicated by the shoulder at 31500 cm^{-1}. ATR-IR (Figure S1B) demonstrate that both samples contain tetrahedral Ti as inferred by DR-UV-Vis, as they present a sharp band a 960 cm^{-1} due to the perturbation of the Si-O-Si bridges antisymmetric stretching as imparted by the Ti substitution.[6] The Raman spectra (Figure S1C) confirm TS-1B contains a fraction of a segregated TiO_2 phase, recognized as anatase from its diagnostic peak at 144 cm^{-1}.[7] TS-1A, instead, is shown to be completely TiO_2-free. The content of tetrahedral Ti and anatase TiO_2 has been quantified from the ATR-IR and Raman spectra through the method described in ref.[1]: in TS-1A tetrahedral Ti accounts for 100% of the Ti quantified by ICP (2.44 wt% TiO_2), corresponding to ca. 2.4 Ti atoms per unit cell. In TS-1B, instead, 89% of Ti is inserted as tetrahedral in the **MFI** framework, whereas the remaining 11% is split into hexacoordinated framework sites (7%) and anatase (4%). On these bases, the content of perfect tetrahedral Ti approaches 2.5 atoms per unit cell.

Two different reference Silicalite-1 materials have been adopted in this work: a defective Silicalite-1 (DS-1), provided by Evonik Industries AG, and an *ad hoc* synthesized, defect-free Silicalite-1 (S-1), prepared according to the verified IZA zeolite synthesis in fluoride medium.[8] Defectivity in all presented materials, to be intended as presence of missing tetrahedral sites and consequent formation of silanol nests, has been checked by transmission IR spectroscopy (collected on a Bruker Vertex 70 spectrophotometer, equipped with MCT detector, on materials outgassed at 400 °C for 2 h prior measurement), as reported in Supplementary Figure 2.

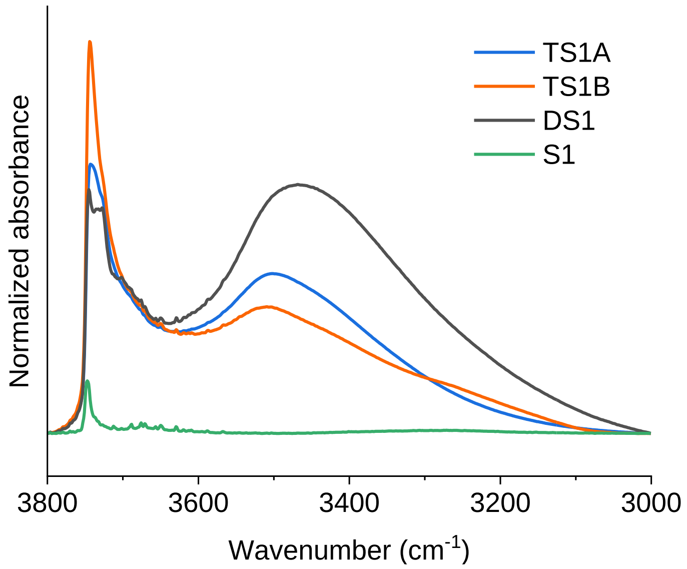

Supplementary Figure 2. Transmission IR spectra of all (Titanium) Silicalite-1 samples considered in this work, reported in the OH stretching modes region. The spectra have been normalized to the intensity framework overtones to allow their quantitative comparison.

The transmission IR spectra of materials in the OH stretching modes region are characterized by a main, sharp signal presenting its maximum at 3745 cm^{-1}, typical for isolated silanol groups, namely not interacting by H-bond with other surrounding species.[1,9] Nonetheless, this band is accompanied by further shoulders for all samples (but defect-free S-1), peaked at lower wavenumbers (e.g. 3735 and 3725 cm^{-1}, clearly visible in the DS-1 sample). These perturbed silanol O-H stretching modes are associated to the so-called chain-terminals, i.e. silanols that accept H-bond from their neighbours. [1,9] The extent or the down-shift increases as the number of silanols in the chain increases.

As a counterpart of the chain-terminals, a broad band, approximately peaking between 3400-3500 cm^{-1}, is formed. This can be associated to the O-H stretching modes of all silanols donating hydrogen bond within silanol chains and silanol nests. Comparing the different samples in terms of intensities, DS-1 owns the larger number of defects, meaning it could not be suitable as a reference for differential studies with TS-1A and TS-1B (vide infra section on Pair Distribution Function), both featuring a different (indeed, lower) degree of defectivity. In this regard, the *ad hoc* prepared S-1 sample features a closely defect free structure, making it optimal as reference for the aforementioned study.

Crystallographic data

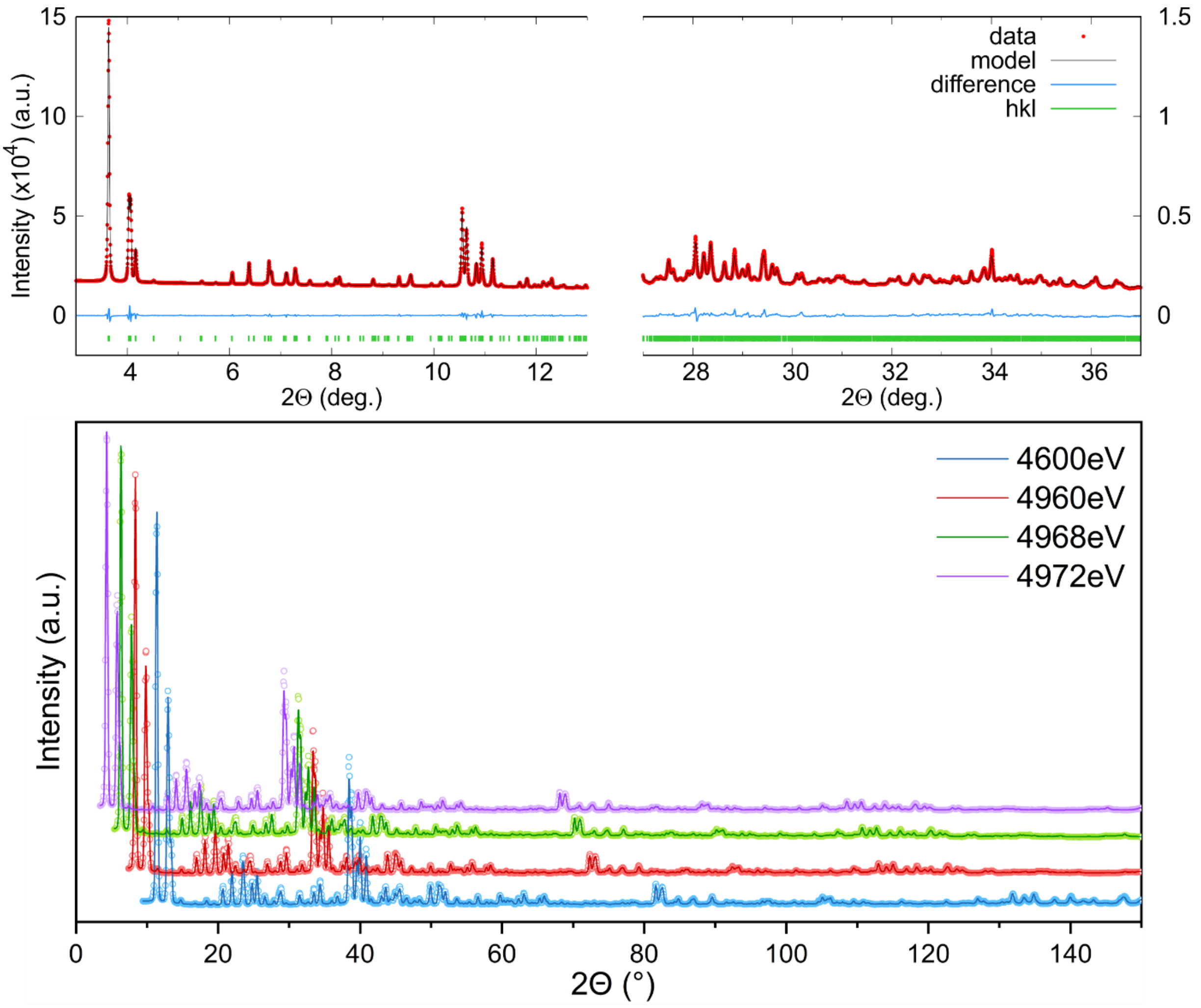

Supplementary Figure 3. Experimental patterns and Rietveld refinement profiles for TS-1A. Observed data represented by dots, calculated models by lines. (Top) Profile fits for the conventional synchrotron powder diffraction data (λ = 0.708597 Å, E = 17.5 keV). (Bottom) Profile fits for the data collected across the Ti K-edge.

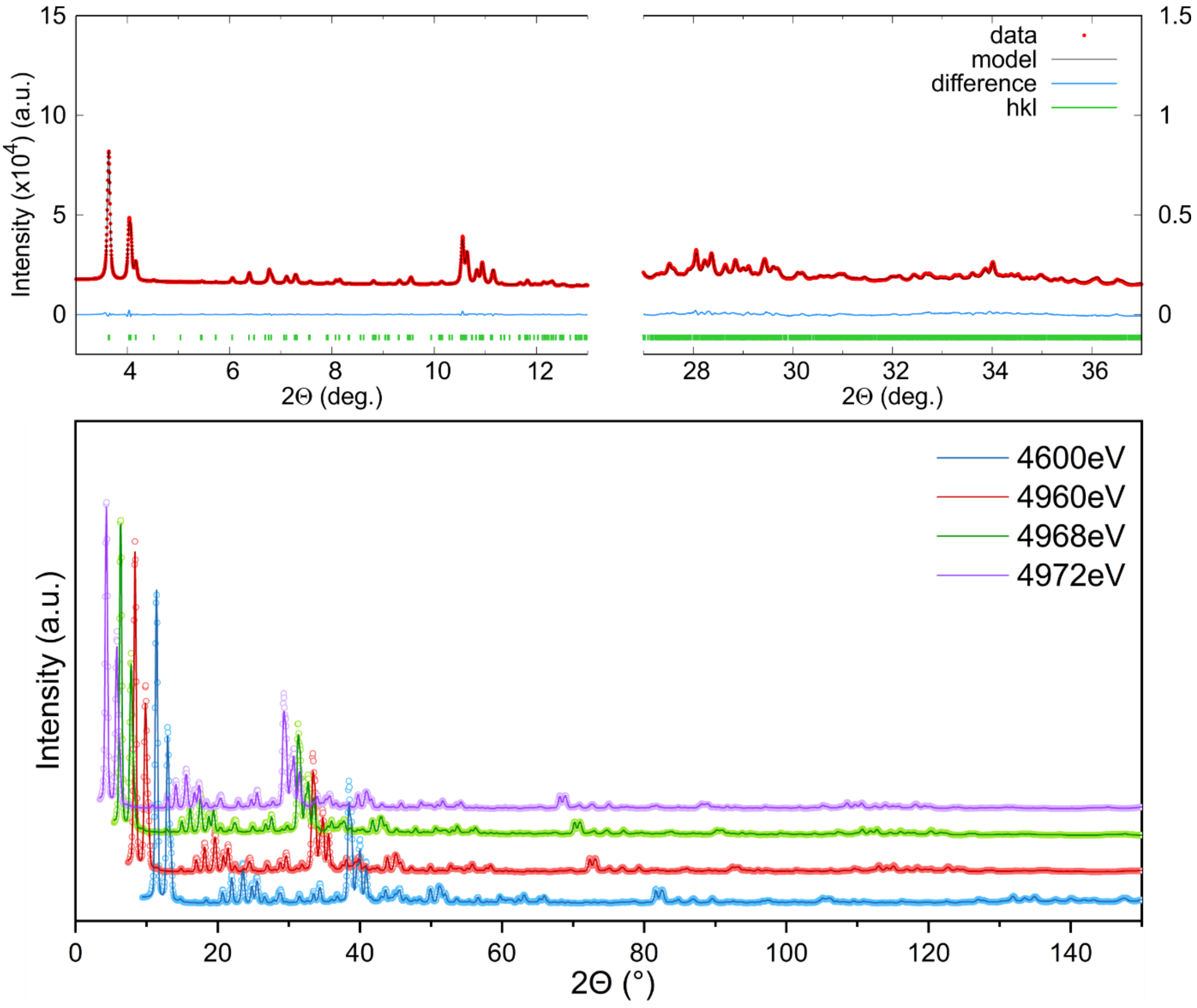

Supplementary Figure 4. Experimental patterns and Rietveld refinement profiles for TS-1B. Observed data represented by dots, calculated models by lines. (Top) Profile fits for the conventional synchrotron powder diffraction data (λ = 0.708597 Å, E = 17.5 keV). (Bottom) Profile fits for the data collected across the Ti K-edge.

Supplementary Table 1. Details of the Rietveld refinement of TS-1A.

Refinement with using conventional powder diffraction data			
Chemical composition		**[**$Si_{93.8}Ti_{2.2}O_{192}$**]**	
Space group		Pnma	
a (Å)		20.1452(1)	
b (Å)		19.9708(1)	
c (Å)		13.4314(1)	
Wavelength (Å)		0.708597	
2θ range (°)		2.9 - 50	
Observations		13082	
Reflections		5084	
**Profile parameters** (cell, peak, *xy*-offset)		9	
Structure parameters (scl, *xyz*, *B*)		121	
Restraints		3	
R_{wp}		2.58	
R_{exp}		1.80	
R_{Bragg}		0.94	
AXRD data collection			
Energy (keV)	2θ range (°)	Observations	Reflections
4.600	9.4 - 160	402	1155
4.960	9.4 - 160	404	1448
4.968	9.4 - 160	401	1451
4.972	9.4 - 160	401	1453
Parameters refined using all three AXRD data sets			
a = 20.1156(3) Å		1	
b = 19.9372(3) Å		1	
c = 13.4091(2) Å		1	
Sample displacement		1	
Peak shape		5	
Scale		1	

Structure coordinates xyz	110
B_{iso}	2
Ti occupancy	12

Supplementary Table 2. Details of the Rietveld refinement of TS-1B.

Refinement with conventional powder diffraction data			
Chemical composition		**[**$Si_{94.2}Ti_{1.8}O_{192}$**]**	
Space group		Pnma	
a (Å)		20.1350(2)	
b (Å)		19.9543(3)	
c (Å)		13.4226(2)	
Wavelength (Å)		0.708597	
2θ range (°)		2.9 - 50	
Observations		13082	
Reflections		5070	
**Profile parameters** (cell, peak, *xy*-offset)		9	
Structure parameters (scl, *xyz*, *B*)		121	
Restraints		3	
R_{wp}		2.17	
R_{exp}		1.77	
R_{Bragg}		0.97	
AXRD data collection			
Energy (keV)	2θ range (°)	Observations	Reflections
4.600	9.4 - 160	402	1155
4.960	9.4 - 160	404	1448
4.968	9.4 - 160	401	1451
4.972	9.4 - 160	401	1453
Parameters refined using all three AXRD data sets			
a = 20.1157(4) Å		1	

b = 19.9338(4) Å	1
c = 13.4080(3) Å	1
Sample displacement	1
Peak shape	5
Scale	1
Structure coordinates xyz	110
B_{iso}	2
Ti occupancy	12

Structural Data for TS-1A

```
data_TS1A
_cell_length_a 20.1174
_cell_length_b 19.93851
_cell_length_c 13.41013
_cell_angle_alpha 90
_cell_angle_beta 90
_cell_angle_gamma 90
_cell_volume 5378.9504
_symmetry_space_group_name_H-M Pnma
loop_
        _symmetry_equiv_pos_as_xyz
        'x, y, z '
        '-x, y+1/2, -z '
        '-x+1/2, -y, z+1/2 '
        '-x+1/2, y+1/2, z+1/2 '
        'x, -y+1/2, z '
        '-x, -y, -z '
        'x+1/2, -y+1/2, -z+1/2 '
        'x+1/2, y, -z+1/2 '
loop_
_atom_site_label
_atom_site_type_symbol
```

_atom_site_symmetry_multiplicity
_atom_site_fract_x
_atom_site_fract_y
_atom_site_fract_z
_atom_site_occupancy
_atom_site_B_iso_or_equiv
T1 Si 8 0.42396 0.05891 0.66526 0.96(1) 2.54607
T1 Ti 8 0.42396 0.05891 0.66526 0.04(1) 2.54607
T2 Si 8 0.311 0.03292 0.82308 0.97(2) 2.54607
T2 Ti 8 0.311 0.03292 0.82308 0.03(2) 2.54607
T3 Si 8 0.27651 0.05813 0.03041 0.93(2) 2.54607
T3 Ti 8 0.27651 0.05813 0.03041 0.07(2) 2.54607
T4 Si 8 0.11796 0.06155 0.02463 0.98(1) 2.54607
T4 Ti 8 0.11796 0.06155 0.02463 0.02(1) 2.54607
T5 Si 8 0.07064 0.0286 0.8171 1.00(1) 2.54607
T5 Ti 8 0.07064 0.0286 0.8171 0.00(1) 2.54607
T6 Si 8 0.18646 0.06088 0.67251 1.00(1) 2.54607
T6 Ti 8 0.18646 0.06088 0.67251 0.00(1) 2.54607
T7 Si 8 0.42667 0.82549 0.67457 0.98(1) 2.54607
T7 Ti 8 0.42667 0.82549 0.67457 0.02(1) 2.54607
T8 Si 8 0.31281 0.87044 0.81677 1.00(0) 2.54607
T8 Ti 8 0.31281 0.87044 0.81677 0.00(0) 2.54607
T9 Si 8 0.27466 0.82862 0.03426 0.94(2) 2.54607
T9 Ti 8 0.27466 0.82862 0.03426 0.06(2) 2.54607
T10 Si 8 0.12009 0.82332 0.03397 1.00(2) 2.54607
T10 Ti 8 0.12009 0.82332 0.03397 0.00(2) 2.54607
T11 Si 8 0.06943 0.8706 0.81769 0.98(2) 2.54607
T11 Ti 8 0.06943 0.8706 0.81769 0.02(2) 2.54607
T12 Si 8 0.18843 0.8239 0.68384 0.98(2) 2.54607
T12 Ti 8 0.18843 0.8239 0.68384 0.02(2) 2.54607
O1 O 8 0.4967 0.05161 0.71189 1 1.18747
O2 O 8 0.37365 0.05317 0.77015 1 1.18747
O3 O 8 0.4135 0.13374 0.60934 1 1.18747

O4 O 8 0.4081 0.01868 0.59921 1 1.18747
O5 O 8 0.30373 0.0603 0.92641 1 1.18747
O6 O 8 0.30875 0.95548 0.81483 1 1.18747
O7 O 8 0.24401 0.05061 0.74152 1 1.18747
O8 O 8 0.29922 0.13251 0.09659 1 1.18747
O9 O 8 0.19179 0.05825 0.03037 1 1.18747
O10 O 8 0.30368 0.01137 0.10027 1 1.18747
O11 O 8 0.10026 0.12754 0.06132 1 1.18747
O12 O 8 0.09381 0.06994 0.92239 1 1.18747
O13 O 8 0.11238 0.05868 0.72655 1 1.18747
O14 O 8 0.08209 0.95177 0.82934 1 1.18747
O15 O 8 0.18749 0.12182 0.6202 1 1.18747
O16 O 8 0.51058 0.85716 0.72233 1 1.18747
O17 O 8 0.38619 0.84149 0.76505 1 1.18747
O18 O 4 0.41872 0.75 0.63177 1 1.18747
O19 O 8 0.30285 0.83633 0.93471 1 1.18747
O20 O 8 0.26869 0.85066 0.77783 1 1.18747
O21 O 4 0.2929 0.75 0.06923 1 1.18747
O22 O 8 0.20104 0.84232 0.03074 1 1.18747
O23 O 4 0.10189 0.75 0.08314 1 1.18747
O24 O 8 0.09039 0.84462 0.89158 1 1.18747
O25 O 8 0.13191 0.84056 0.71896 1 1.18747
O26 O 4 0.19078 0.75 0.64474 1 1.18747

Structural Data for TS-1B

data_TS1B
_cell_length_a 20.11951
_cell_length_b 19.93614
_cell_length_c 13.40944
_cell_angle_alpha 90
_cell_angle_beta 90
_cell_angle_gamma 90
_cell_volume 5378.59837

```
_symmetry_space_group_name_H-M Pnma
loop_
        _symmetry_equiv_pos_as_xyz
        'x, y, z '
        '-x, y+1/2, -z '
        '-x+1/2, -y, z+1/2 '
        '-x+1/2, y+1/2, z+1/2 '
        'x, -y+1/2, z '
        '-x, -y, -z '
        'x+1/2, -y+1/2, -z+1/2 '
        'x+1/2, y, -z+1/2 '
loop_
_atom_site_label
_atom_site_type_symbol
_atom_site_symmetry_multiplicity
_atom_site_fract_x
_atom_site_fract_y
_atom_site_fract_z
_atom_site_occupancy
_atom_site_B_iso_or_equiv
T1 Si   8 0.42337 0.05726 0.66434 1.00(1) 3.11512
T1 Ti   8 0.42337 0.05726 0.66434 0.00(1) 3.11512
T2 Si   8 0.30885 0.03277 0.82512 0.96(2) 3.11512
T2 Ti   8 0.30885 0.03277 0.82512 0.04(2) 3.11512
T3 Si   8 0.27543 0.05924 0.0289 0.94(2) 3.11512
T3 Ti   8 0.27543 0.05924 0.0289 0.06(2) 3.11512
T4 Si   8 0.1171 0.05959 0.02442 0.99(1) 3.11512
T4 Ti   8 0.1171 0.05959 0.02442 0.01(1) 3.11512
T5 Si   8 0.06765 0.02995 0.81398 1.00(1) 3.11512
T5 Ti   8 0.06765 0.02995 0.81398 0.00(1) 3.11512
T6 Si   8 0.18878 0.06218 0.66938 1.00(1) 3.11512
T6 Ti   8 0.18878 0.06218 0.66938 0.00(1) 3.11512
T7 Si   8 0.42575 0.82452 0.67561 1.00(1) 3.11512
```

T7 Ti 8 0.42575 0.82452 0.67561 0.00(1) 3.11512
T8 Si 8 0.31301 0.86692 0.81718 0.99(1) 3.11512
T8 Ti 8 0.31301 0.86692 0.81718 0.01(1) 3.11512
T9 Si 8 0.27836 0.82789 0.03609 0.95(2) 3.11512
T9 Ti 8 0.27836 0.82789 0.03609 0.05(2) 3.11512
T10 Si 8 0.12056 0.82351 0.0312 0.96(2) 3.11512
T10 Ti 8 0.12056 0.82351 0.0312 0.04(2) 3.11512
T11 Si 8 0.07216 0.86605 0.81435 1.00(1) 3.11512
T11 Ti 8 0.07216 0.86605 0.81435 0.00(1) 3.11512
T12 Si 8 0.18772 0.82382 0.68346 0.98(2) 3.11512
T12 Ti 8 0.18772 0.82382 0.68346 0.02(2) 3.11512
O1 O 8 0.49968 0.0474 0.7254 1 1.70372
O2 O 8 0.3758 0.05452 0.77144 1 1.70372
O3 O 8 0.41589 0.12968 0.6149 1 1.70372
O4 O 8 0.39923 -0.01064 0.56554 1 1.70372
O5 O 8 0.30484 0.06086 0.92226 1 1.70372
O6 O 8 0.30679 0.93821 0.81954 1 1.70372
O7 O 8 0.24149 0.0535 0.73224 1 1.70372
O8 O 8 0.29944 0.13191 0.09788 1 1.70372
O9 O 8 0.20296 0.05638 0.02151 1 1.70372
O10 O 8 0.30691 0.00226 0.10747 1 1.70372
O11 O 8 0.09816 0.13034 0.0628 1 1.70372
O12 O 8 0.09411 0.07159 0.9199 1 1.70372
O13 O 8 0.11437 0.05647 0.71946 1 1.70372
O14 O 8 0.07691 0.95857 0.82327 1 1.70372
O15 O 8 0.18925 0.12906 0.61275 1 1.70372
O16 O 8 0.51352 0.85497 0.73171 1 1.70372
O17 O 8 0.38779 0.84167 0.77013 1 1.70372
O18 O 4 0.42081 0.75 0.64017 1 1.70372
O19 O 8 0.30228 0.83654 0.91724 1 1.70372
O20 O 8 0.26665 0.8581 0.77136 1 1.70372
O21 O 4 0.29358 0.75 0.07517 1 1.70372
O22 O 8 0.18754 0.83728 0.0356 1 1.70372

O23 O 4 0.09892 0.75 0.08848 1 1.70372
O24 O 8 0.09272 0.84568 0.90517 1 1.70372
O25 O 8 0.13492 0.83467 0.72451 1 1.70372
O26 O 4 0.19067 0.75 0.62769 1 1.70372

Table S3. Linear Regression Coefficients of Unit Cell Parameters

	m*	q*	R^2
a	2.28384 Å	20.08841 Å	0.99324
b	3.12303 Å	19.89479 Å	0.98899
c	1.96537 Å	13.38359 Å	0.99511
V	2302.123 $Å^3$	5347.450 $Å^3$	0.99570

*Y=mx+q, where Y=a, b, c, and V

Pair Distribution Function

The radially-averaged distribution of distances between titanium atoms that belong to T3 and/or T9 sites(s) has been analyzed by the pair distribution function (PDF) method based on 50keV total scattering data (Supplementary Figure 5A). The G(r) function of each sample was correctly reproduced by the respective structural model derived from AXRD, after relaxing atomic positions to account for correlated atomic displacements and peak broadening. The G(r) observed for the reference defect-free Ti-free silicalite-1 (S-1) was subtracted from the G(r) for TS1A and TS-1B (Supplementary Figure 5B). The resulting Differential Pair Distribution Function (dPDF) highlighted the signal from a diluted dopant by excluding any interatomic distances shared by all samples.[10] The real-space distance 1.84 Å corresponds clearly to Ti-O bond length and negative peaks 1.62 Å and 3.11 Å indicate the deficiency of Si-O and Si-O-Si pairs respectively. This confirms that titanium substitutes silicon as a heteroatom in the TS-1 structure. The remaining peaks either overlap with those generated by ΔG(r) calculated by subtracting the reference S1 from the defected silicalite-1 (DS-1), hence can be assigned to the defect sites, or result from peak broadening of G(r) from both TS-1, which is associated with local disorder in the structures.

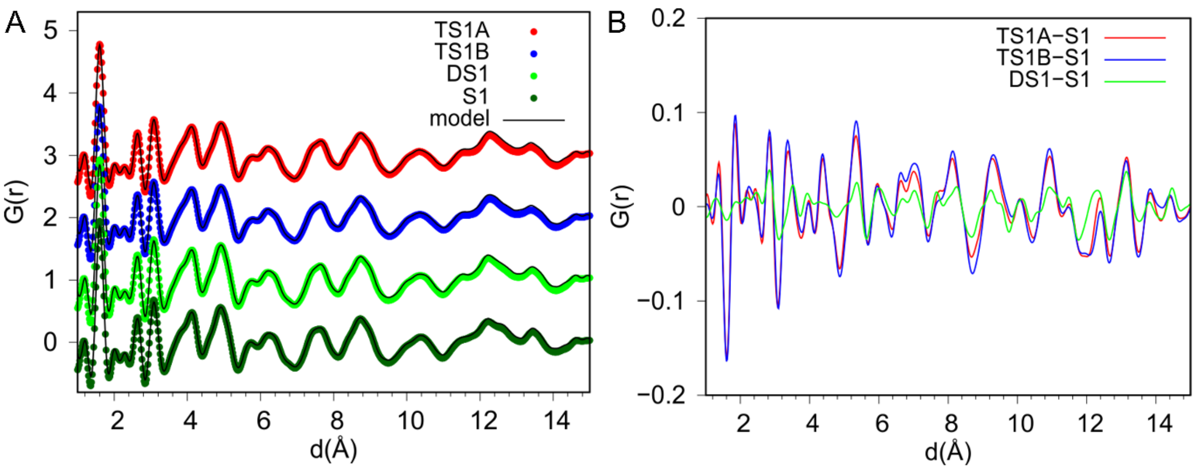

Supplementary Figure 5. (A) Pair distribution functions of titanium silicalite-1 (TS-1A and TS-1B), defected titanium-free silicalite-1 (DS-1), defect-free titanium-free silicalite-1 (S-1), and refined P1 models. (B) Differential pair distribution function generated by subtracting the PDF of S-1 from those of TS-1A, TS-1B, and DS-1.

DFT calculations

Supplementary Table 4. Cell parameters (a, b , c) and volumes (V), minimal Ti-Ti distances (dTi-Ti), average Ti-O distance (<dTi-O>) multiplicities of Ti pair models and relative energies (ΔE) for the 22 models (including SDA) featuring 2 Ti at T3/T9 sites. For sites labelling refer to the cif of DFT optimized Silicalite-1 (vide infra). Cell parameters and distances in Å, cell volume in Å3, relative energies in kJ/mol.

Ti-sub. sites	M	a	b	c	V	d_{Ti-Ti}	$\langle d_{Ti-O} \rangle$	ΔE
T9(g)-T9(h)	4	20.436	20.273	13.527	5604.5	9.646	1.89±0.03	5.5
T9(f)-T9(h)	4	20.390	20.294	13.501	5586.5	11.897	1.89±0.03	4.1
T9(f)-T9(g)	4	20.462	20.311	13.517	5617.8	13.517	1.89±0.04	18.2
T9(d)-T9(h)	4	20.455	20.301	13.523	5615.6	12.168	1.89±0.04	0.0
T9(d)-T9(g)	4	20.448	20.297	13.547	5622.3	12.226	1.90±0.04	9.2
T9(d)-T9(f)	4	20.476	20.282	13.528	5618.2	12.177	1.89±0.03	23.4
T9(d)-T9(e)	4	20.451	20.280	13.540	5615.6	3.363	1.89±0.06	56.2
T3(h)-T9(h)	8	20.393	20.245	13.576	5605.1	4.733	1.90±0.04	36.2
T3(h)-T9(g)	8	20.361	20.236	13.581	5595.4	7.180	1.89±0.04	38.7
T3(h)-T9(f)	8	20.391	20.273	13.562	5606.4	13.562	1.89±0.03	21.2
T3(h)-T9(e)	8	20.415	20.283	13.566	5617.2	10.583	1.89±0.04	22.7
T3(h)-T9(d)	8	20.406	20.275	13.584	5620.1	9.444	1.89±0.04	38.9
T3(h)-T9(c)	8	20.388	20.276	13.567	5608.2	12.067	1.89±0.04	12.1

T3(h)-T9(b)	8	20.364	20.251	13.568	5595.2	8.595	1.89±0.04	28.6
T3(h)-T9(a)	8	20.398	20.222	13.591	5606.2	7.933	1.89±0.04	53.3
T3(g)-T3(h)	4	20.359	20.227	13.588	5595.6	7.393	1.89±0.05	44.9
T3(f)-T3(h)	4	20.393	20.201	13.595	5600.7	13.595	1.89±0.03	17.9
T3(f)-T3(g)	4	20.394	20.173	13.618	5602.8	13.379	1.89±0.03	11.8
T3(d)-T3(h)	4	20.348	20.236	13.596	5598.2	8.811	1.89±0.04	20.3
T3(d)-T3(g)	4	20.319	20.219	13.590	5583.3	11.661	1.89±0.04	24.2
T3(d)-T3(f)	4	20.353	20.212	13.628	5606.5	12.302	1.89±0.04	22.6
T3(d)-T3(e)	4	20.343	20.159	13.649	5597.2	7.713	1.89±0.04	66.3

Supplementary Table 5. Cell parameters (a, b , c) and volumes (V), minimal Ti-Ti distances (dTi-Ti), average Ti-O distance (<dTi-O>) multiplicities of Ti pair models and relative energies (ΔE) for the 22 models (not including SDA) featuring 2 Ti at T3/T9 sites. For sites labelling refer to the cif of DFT optimized Silicalite-1 (vide infra). Cell parameters and distances in Å, cell volume in Å3, relative energies in kJ/mol.

**Ti-sub. sites**	**M**	**a**	**b**	**c**	**V**	d_{Ti-Ti}	<d_{Ti-O}>	**ΔE**
T9(g)-T9(h)	4	20.229	19.950	13.375	5398.0	9.649	1.799±0.006	20.8
T9(f)-T9(h)	4	20.215	19.961	13.386	5401.4	11.925	1.798±0.007	15.3
T9(f)-T9(g)	4	20.211	19.968	13.376	5398.0	13.376	1.800±0.010	18.7
T9(d)-T9(h)	4	20.222	19.958	13.386	5402.4	11.525	1.799±0.006	14.3
T9(d)-T9(g)	4	20.220	19.963	13.380	5401.2	11.515	1.799±0.007	15.2
T9(d)-T9(f)	4	20.227	19.957	13.388	5404.3	12.048	1.797±0.007	18.9
T9(d)-T9(e)	4	20.229	19.935	13.376	5394.0	3.259	1.801±0.010	22.1
T3(h)-T9(h)	8	20.243	19.916	13.393	5399.1	4.492	1.803±0.009	7.9
T3(h)-T9(g)	8	20.245	19.925	13.382	5398.2	6.948	1.803±0.008	1.0
T3(h)-T9(f)	8	20.240	19.927	13.391	5401.0	13.391	1.800±0.008	12.8
T3(h)-T9(e)	8	20.238	19.928	13.398	5403.7	10.645	1.800±0.005	10.1
T3(h)-T9(d)	8	20.240	19.931	13.397	5404.3	9.482	1.800±0.005	8.0
T3(h)-T9(c)	8	20.237	19.928	13.388	5399.2	12.212	1.800±0.008	12.9
T3(h)-T9(b)	8	20.235	19.919	13.389	5396.6	8.485	1.802±0.009	8.0
T3(h)-T9(a)	8	20.239	19.917	13.397	5400.3	7.660	1.804±0.006	3.6
T3(g)-T3(h)	4	20.248	19.888	13.402	5396.9	7.131	1.806±0.008	1.2
T3(f)-T3(h)	4	20.253	19.891	13.407	5400.8	13.407	1.803±0.005	4.2
T3(f)-T3(g)	4	20.262	19.901	13.407	5406.1	13.407	1.802±0.006	5.9
T3(d)-T3(h)	4	20.255	19.900	13.396	5399.5	9.367	1.802±0.006	5.7
T3(d)-T3(g)	4	20.251	19.894	13.405	5400.5	11.283	1.802±0.006	7.6
T3(d)-T3(f)	4	20.249	19.900	13.401	5400.0	12.031	1.802±0.007	6.2
T3(d)-T3(e)	4	20.256	19.888	13.402	5399.0	7.798	1.804±0.004	0.0

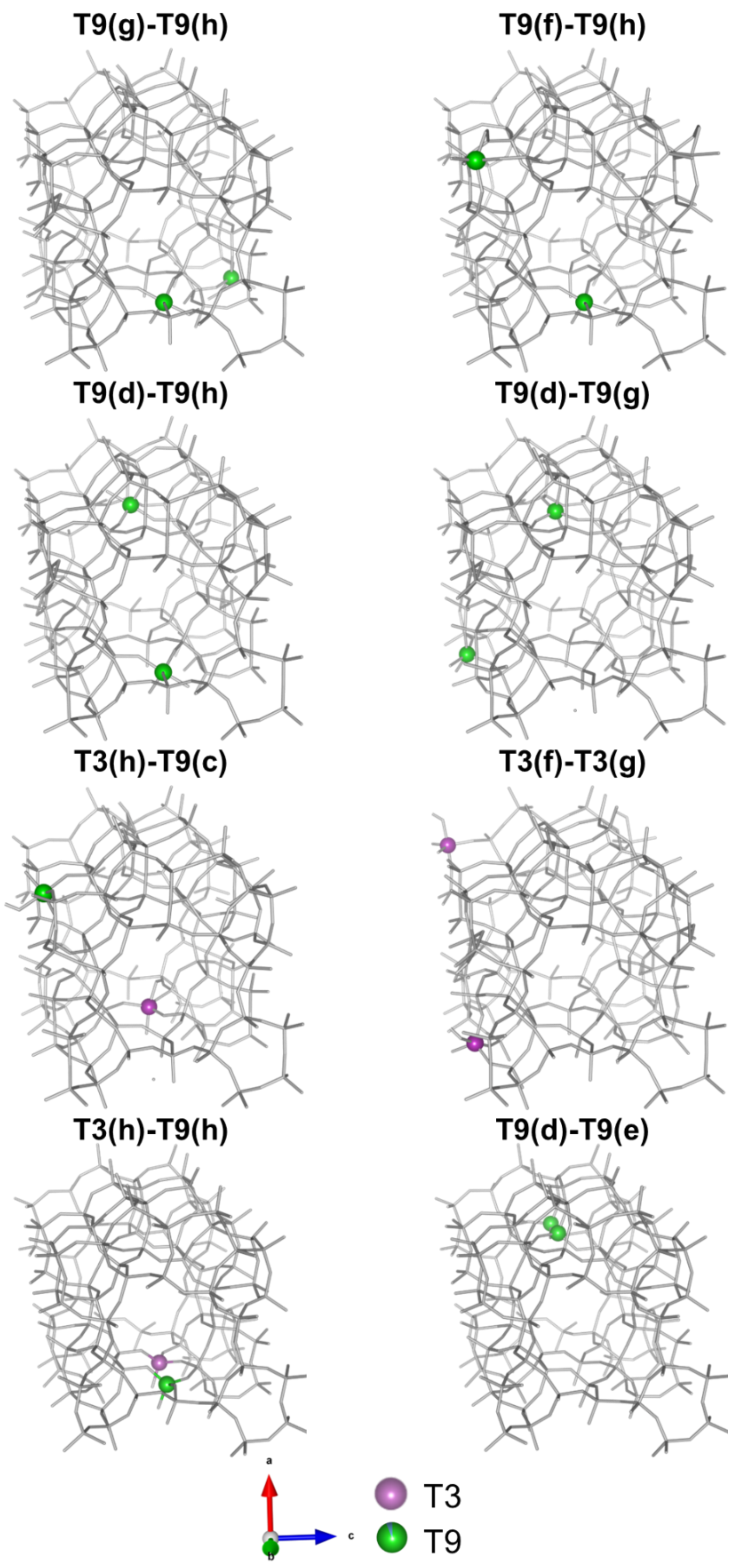

Supplementary Figure 6. Graphical representation of DFT optimized models with Ti substituted at sites: T3(d)-T3(e), T3(h)-T9(g), T3(g)-T3(h), T3(h)-T9(a) and T9(d)-T9(e). Both Si and O atoms as grey sticks, Ti at T3 as magenta sphere, Ti at T9 as green sphere. TPA^+ omitted for clarity.

Structural Data for DFT optimized Silicalite-1 (with $TPA^{+}OH^{-}$)

```
data_S1-DFT
_cell_length_a 20.448540
_cell_length_b 20.198196
_cell_length_c 13.567860
_cell_angle_alpha 90
_cell_angle_beta 90
_cell_angle_gamma 90
_cell_volume
_symmetry_space_group_name_H-M P1
loop_
    _symmetry_equiv_pos_as_xyz
     'x, y, z '
loop_
_atom_site_label
_atom_site_type_symbol
_atom_site_fract_x
_atom_site_fract_y
_atom_site_fract_z
T1(a) Si 0.421724152864 0.063246950104 0.658756290159
T1(b) Si 0.305644945189 0.028612190450 0.807202337414
T1(c) Si 0.280843446412 0.059406776580 0.029949371220
T1(d) Si 0.122148756102 0.059121618852 0.025366805104
T1(e) Si 0.068261256344 0.031083858821 0.808124192135
T1(f) Si 0.183589903088 0.062194504296 0.670459345320
T1(g) Si 0.419524013348 0.827836983531 0.672058136972
T1(h) Si 0.303617507402 0.867823528463 0.815717980993
T2(a) Si 0.266802018354 0.826831518785 0.033429625107
T2(b) Si 0.120114790068 0.818807028534 0.018988148153
T2(c) Si 0.067173171292 0.872135792033 0.810533430851
T2(d) Si 0.183722977861 0.825887814679 0.677735901166
T2(e) Si 0.578275851401 0.936753056852 0.341243703436
T2(f) Si 0.694355057109 0.971387820378 0.192797660427
```

346	T2(g)	Si	0.719156551485	0.940593233519	0.970050622559
347	T2(h)	Si	0.877851249053	0.940878391199	0.974633197962
348	T3(a)	Si	0.931738742698	0.968916153339	0.191875805698
349	T3(b)	Si	0.816410102589	0.937805506096	0.329540645393
350	T3(c)	Si	0.580475993582	0.172163024465	0.327941863868
351	T3(d)	Si	0.696382501719	0.132176484375	0.184282003426
352	T3(e)	Si	0.733197991979	0.173168491498	0.966570368813
353	T3(f)	Si	0.879885229987	0.181192987017	0.981011855753
354	T3(g)	Si	0.932826829969	0.127864212690	0.189466578281
355	T3(h)	Si	0.816277028541	0.174112198476	0.322264095952
356	T4(a)	Si	0.079318267691	0.935018511836	0.159218053798
357	T4(b)	Si	0.195055373714	0.969945199824	0.306611581716
358	T4(c)	Si	0.219947372565	0.940481457869	0.530862528099
359	T4(d)	Si	0.378032844565	0.940260276482	0.525562198431
360	T4(e)	Si	0.430830053001	0.969006603392	0.308125937119
361	T4(f)	Si	0.316734532035	0.936591655225	0.169074971985
362	T4(g)	Si	0.078922708008	0.171847197695	0.171357346125
363	T4(h)	Si	0.196234653335	0.130669218172	0.313860624159
364	T5(a)	Si	0.233031284292	0.171695385504	0.531701333482
365	T5(b)	Si	0.380413286733	0.180885488224	0.518342417426
366	T5(c)	Si	0.433679444259	0.127863326883	0.310543579791
367	T5(d)	Si	0.317122113765	0.173454586410	0.178103179389
368	T5(e)	Si	0.920681727977	0.064981501115	0.840781939827
369	T5(f)	Si	0.804944627748	0.030054804533	0.693388417820
370	T5(g)	Si	0.780052633533	0.059518556256	0.469137469844
371	T5(h)	Si	0.621967157513	0.059739729207	0.474437813760
372	T6(a)	Si	0.569169947928	0.030993402822	0.691874067576
373	T6(b)	Si	0.683265472361	0.063408354439	0.830925016628
374	T6(c)	Si	0.921077289410	0.828152806874	0.828642656248
375	T6(d)	Si	0.803765349859	0.869330795295	0.686139373453
376	T6(e)	Si	0.766968711859	0.828304631929	0.468298665243
377	T6(f)	Si	0.619586711007	0.819114512756	0.481657577208
378	T6(g)	Si	0.566320556382	0.872136686613	0.689456409279

379	T6(h)	Si	0.682877885683	0.826545423136	0.821896806925
380	T7(a)	Si	0.578904973494	0.565039303530	0.341186451268
381	T7(b)	Si	0.694613954292	0.528714566816	0.193073208892
382	T7(c)	Si	0.719328201955	0.560205214396	0.970505922710
383	T7(d)	Si	0.878185659915	0.559520398832	0.974387279254
384	T7(e)	Si	0.931675466082	0.531998631209	0.190767209759
385	T7(f)	Si	0.816403480058	0.563301138572	0.330628827206
386	T7(g)	Si	0.579873018978	0.327851300468	0.329170772104
387	T7(h)	Si	0.695939713080	0.367900770554	0.184723771877
388	T8(a)	Si	0.732951016087	0.327717573446	0.968160496593
389	T8(b)	Si	0.879888519097	0.318205779036	0.981382647278
390	T8(c)	Si	0.933658755261	0.372446172341	0.188922103125
391	T8(d)	Si	0.816276926842	0.326805247558	0.322025074758
392	T8(e)	Si	0.421095018779	0.434960701733	0.658813559301
393	T8(f)	Si	0.305386042842	0.471285445299	0.806926791174
394	T8(g)	Si	0.280671810829	0.439794780044	0.029494076649
395	T8(h)	Si	0.121814353279	0.440479589633	0.025612708165
396	T9(a)	Si	0.068324534241	0.468001382371	0.809232792821
397	T9(b)	Si	0.183596515453	0.436698867018	0.669371166573
398	T9(c)	Si	0.420126980767	0.672148703785	0.670829217931
399	T9(d)	Si	0.304060293987	0.632099241368	0.815276224519
400	T9(e)	Si	0.267048966792	0.672282430966	0.031839499184
401	T9(f)	Si	0.120111462691	0.681794242644	0.018617345035
402	T9(g)	Si	0.066341241443	0.627553836937	0.811077891834
403	T9(h)	Si	0.183723072938	0.673194761488	0.677974924395
404	T10(a)	Si	0.920136783893	0.435001128561	0.841332481054
405	T10(b)	Si	0.805249622379	0.472130544886	0.692651280747
406	T10(c)	Si	0.780182622622	0.441047868072	0.469894772116
407	T10(d)	Si	0.621746214585	0.441749172500	0.475079538394
408	T10(e)	Si	0.568510314350	0.469173757102	0.692148995656
409	T10(f)	Si	0.683532472754	0.438237650803	0.830635506852
410	T10(g)	Si	0.921476800275	0.672524405214	0.826505574284
411	T10(h)	Si	0.804062804860	0.632638078079	0.684869528997

412	T11(a)	Si	0.766674390871	0.673185942982	0.467800489171
413	T11(b)	Si	0.619588324364	0.681518691026	0.480942574253
414	T11(c)	Si	0.566200483947	0.628479510893	0.688975196037
415	T11(d)	Si	0.682965426397	0.673655546891	0.821870265819
416	T11(e)	Si	0.079863218274	0.564998877459	0.158667495434
417	T11(f)	Si	0.194750363669	0.527869475799	0.307348734133
418	T11(g)	Si	0.219817394014	0.558952143815	0.530105239809
419	T11(h)	Si	0.378253802492	0.558250818088	0.524920447278
420	T12(a)	Si	0.431489684941	0.530826249305	0.307850992485
421	T12(b)	Si	0.316467517969	0.561762366297	0.169364496413
422	T12(c)	Si	0.078523205236	0.327475600875	0.173494438180
423	T12(d)	Si	0.195937197032	0.367361950305	0.315130457316
424	T12(e)	Si	0.233325601036	0.326814071400	0.532199484436
425	T12(f)	Si	0.380411670388	0.318481326877	0.519057448234
426	T12(g)	Si	0.433799516283	0.371520517253	0.311024862602
427	T12(h)	Si	0.317034584049	0.326344465238	0.178129744750
428	O1	O	0.369996361246	0.055413469936	0.750072101540
429	O2	O	0.309686095839	0.059302080577	0.918177360276
430	O3	O	0.201610022050	0.055668320220	0.031250483282
431	O4	O	0.097675572916	0.058887444928	0.911677981185
432	O5	O	0.110922575949	0.063296997425	0.718970078612
433	O6	O	0.238981747377	0.055531400983	0.755827850052
434	O7	O	0.370004224507	0.840131858083	0.764426661500
435	O8	O	0.303056346223	0.840322039077	0.927336644865
436	O9	O	0.193674350536	0.857414473233	0.041358538148
437	O10	O	0.087291180593	0.854160377169	0.920915743905
438	O11	O	0.111073336219	0.837064463123	0.723768917169
439	O12	O	0.238843699677	0.840515347465	0.760188912150
440	O13	O	0.303338930584	0.948270564168	0.812135231886
441	O14	O	0.074117784042	0.951715593705	0.796922223925
442	O15	O	0.417435587426	0.135735855668	0.609807263489
443	O16	O	0.413902524708	0.000609098704	0.582993109333
444	O17	O	0.397779647652	0.868727841848	0.573397903354

445	O18	O	0.188829024913	0.132414399618	0.614247022559
446	O19	O	0.188117873002	0.000524614109	0.593579602089
447	O20	O	0.195327988526	0.870656404186	0.578785910055
448	O21	O	0.993400800346	0.055604524231	0.791504364432
449	O22	O	0.991998429115	0.850451928365	0.785579401881
450	O23	O	0.417869853496	0.750053042925	0.640008902052
451	O24	O	0.188606118398	0.749542629545	0.640014182089
452	O25	O	0.278813109728	0.749465405802	0.063056556052
453	O26	O	0.158232408240	0.750322013213	0.974318194019
454	O27	O	0.437312382322	0.249492463698	0.551981737230
455	O28	O	0.630003636450	0.944586534693	0.249927881147
456	O29	O	0.690313902357	0.940697924255	0.081822638213
457	O30	O	0.798389980928	0.944331690528	0.968749510505
458	O31	O	0.902324435524	0.941112569890	0.088322016692
459	O32	O	0.889077430369	0.936703009848	0.281029917666
460	O33	O	0.761018257059	0.944468605444	0.244172142726
461	O34	O	0.629995789054	0.159868147982	0.235573348833
462	O35	O	0.696943640671	0.159677967498	0.072663335817
463	O36	O	0.806325660355	0.142585540377	0.958641457061
464	O37	O	0.912708825628	0.145839632307	0.079084266281
465	O38	O	0.888926665576	0.162935548729	0.276231078623
466	O39	O	0.761156304827	0.159484660422	0.239811092892
467	O40	O	0.696661072981	0.051729446516	0.187864768630
468	O41	O	0.925882222413	0.048284414014	0.203077762072
469	O42	O	0.582564409591	0.864264146739	0.390192721446
470	O43	O	0.586097479590	0.999390906173	0.417006893475
471	O44	O	0.602220356974	0.131272158613	0.426602091958
472	O45	O	0.811170977639	0.867585613666	0.385752978207
473	O46	O	0.811882128920	0.999475397293	0.406420389612
474	O47	O	0.804672002519	0.129343609172	0.421214077084
475	O48	O	0.006599197434	0.944395492414	0.208495617518
476	O49	O	0.008001568762	0.149548077734	0.214420605497
477	O50	O	0.582130139834	0.249946964744	0.359991103122

478	O51	O	0.811393877098	0.250457381139	0.359985819569
479	O52	O	0.721186885078	0.250534600397	0.936943430298
480	O53	O	0.841767594836	0.249677994619	0.025681802657
481	O54	O	0.562687633366	0.750507557190	0.448018234069
482	O55	O	0.131361024552	0.940828260999	0.250405344815
483	O56	O	0.191833266825	0.940971165068	0.418732893456
484	O57	O	0.298962298986	0.947964366095	0.531964653969
485	O58	O	0.400955854751	0.940855775392	0.411246666505
486	O59	O	0.389329604881	0.936916246337	0.217879808769
487	O60	O	0.262390772847	0.944309409251	0.255789247678
488	O61	O	0.131391901163	0.160167882518	0.260148739986
489	O62	O	0.195425306442	0.156596129258	0.426722355382
490	O63	O	0.306521083784	0.142197014793	0.536488562854
491	O64	O	0.414408782656	0.145947043721	0.421104973621
492	O65	O	0.390214664485	0.163363551839	0.223593672348
493	O66	O	0.262688677911	0.156305999001	0.260691989289
494	O67	O	0.194703198786	0.050318846876	0.307416654432
495	O68	O	0.424605745799	0.048427473592	0.296953127022
496	O69	O	0.081128247361	0.862510347409	0.109988774184
497	O70	O	0.090685906925	0.996410272259	0.082742289197
498	O71	O	0.097786329352	0.129037639233	0.073335293783
499	O72	O	0.312550512775	0.865450490821	0.114823802641
500	O73	O	0.310244123879	0.996436031941	0.089102487069
501	O74	O	0.306680905561	0.128500515462	0.079022789976
502	O75	O	0.505466577717	0.944156649336	0.291670278850
503	O76	O	0.509087101593	0.149459436303	0.286588927716
504	O77	O	0.077522033583	0.249889341177	0.140043205959
505	O78	O	0.309744214878	0.249914698961	0.140769381170
506	O79	O	0.222278840973	0.249263791341	0.562328099568
507	O80	O	0.343172656483	0.249895986379	0.474085723452
508	O81	O	0.063903710754	0.750190281508	0.051948244027
509	O82	O	0.868638975736	0.059171747294	0.749594647299
510	O83	O	0.808166740154	0.059028843328	0.581267106839

511	O84	O	0.701037705313	0.052035654526	0.468035335712
512	O85	O	0.599044138271	0.059144231534	0.588753338242
513	O86	O	0.610670399329	0.063083758460	0.782120186544
514	O87	O	0.737609233085	0.055690603126	0.744210748276
515	O88	O	0.868608102666	0.839832135850	0.739851257619
516	O89	O	0.804574683078	0.843403885288	0.573277645465
517	O90	O	0.693478906641	0.857802971973	0.463511430985
518	O91	O	0.585591218111	0.854052971345	0.578895015868
519	O92	O	0.609785330317	0.836636458063	0.776406323503
520	O93	O	0.737311320569	0.843694020580	0.739308001122
521	O94	O	0.805296803669	0.949681162045	0.692583346954
522	O95	O	0.575394257756	0.951572535948	0.703046871231
523	O96	O	0.918871765525	0.137489669533	0.890011216979
524	O97	O	0.909314094449	0.003589740826	0.917257710553
525	O98	O	0.902213675324	0.870962371001	0.926664705363
526	O99	O	0.687449494957	0.134549499703	0.885176217716
527	O100	O	0.689755879227	0.003563978536	0.910897502075
528	O101	O	0.693319092366	0.871499491440	0.920977203922
529	O102	O	0.494533427350	0.055843365883	0.708329704227
530	O103	O	0.490912901782	0.850540575643	0.713411070368
531	O104	O	0.922477960451	0.750110665150	0.859956798228
532	O105	O	0.690255801955	0.750085313431	0.859230615410
533	O106	O	0.777721149681	0.750736227968	0.437671908798
534	O107	O	0.656827354985	0.750104024052	0.525914283203
535	O108	O	0.936096290557	0.249809735746	0.948051750799
536	O109	O	0.630105570011	0.555368267042	0.249797619079
537	O110	O	0.690535077287	0.559548467606	0.082252968884
538	O111	O	0.798600185456	0.558167625044	0.968672516808
539	O112	O	0.902424070970	0.562224880088	0.088357629169
540	O113	O	0.889032220110	0.562806912632	0.281333542091
541	O114	O	0.761244398705	0.555488772492	0.244825979948
542	O115	O	0.630636734735	0.340487768125	0.239030057651
543	O116	O	0.694106785228	0.340393927968	0.072805366720

544	O117	O	0.806308105713	0.357864048180	0.964610143604
545	O118	O	0.916143394878	0.351165843786	0.078693324452
546	O119	O	0.888621464965	0.339970067342	0.276318508431
547	O120	O	0.761106622825	0.340540871348	0.239253969233
548	O121	O	0.696715820103	0.448379228274	0.187858174686
549	O122	O	0.926002229817	0.452331172784	0.197548441759
550	O123	O	0.582906931947	0.638798392153	0.386326545174
551	O124	O	0.589076482326	0.505728654470	0.421937695679
552	O125	O	0.598650830949	0.371550833084	0.426409149947
553	O126	O	0.811280705195	0.634458043372	0.384954085250
554	O127	O	0.811410698734	0.503405521176	0.411009422628
555	O128	O	0.804898532971	0.371585926090	0.420934613285
556	O129	O	0.006888336703	0.555705291482	0.207870210814
557	O130	O	0.008259904869	0.350543301268	0.218632483059
558	O131	O	0.369894447667	0.444631755965	0.750202412621
559	O132	O	0.309464924440	0.440451543143	0.917747027225
560	O133	O	0.201399825934	0.441832295399	0.031327460653
561	O134	O	0.097575935817	0.437775128284	0.911642360409
562	O135	O	0.110967780360	0.437193103181	0.718666459442
563	O136	O	0.238755606836	0.444511241885	0.755174000918
564	O137	O	0.369363286156	0.659512227741	0.760969954223
565	O138	O	0.305893201216	0.659606087727	0.927194626898
566	O139	O	0.193691874696	0.642135957484	0.035389850713
567	O140	O	0.083856599097	0.648834168834	0.921306667293
568	O141	O	0.111378539690	0.660029945224	0.723681504305
569	O142	O	0.238893388330	0.659459144213	0.760746015105
570	O143	O	0.303284176587	0.551620782777	0.812141828394
571	O144	O	0.073997774858	0.547668839648	0.802451540840
572	O145	O	0.417093076684	0.361201626814	0.613673432207
573	O146	O	0.410923505546	0.494271321919	0.578062282659
574	O147	O	0.401349176425	0.628449158063	0.573590852529
575	O148	O	0.188719290419	0.365541964015	0.615045904476
576	O149	O	0.188589310053	0.496594492503	0.588990584963

577	O150	O	0.195101465718	0.628414085189	0.579065382802
578	O151	O	0.993111667180	0.444294719692	0.792129785987
579	O152	O	0.991740092075	0.649456706025	0.781367509357
580	O153	O	0.868795368349	0.444132029395	0.750006264997
581	O154	O	0.808076350507	0.440760629712	0.582111020839
582	O155	O	0.700997478382	0.445269434691	0.467999343810
583	O156	O	0.598971265919	0.441070359081	0.589433899097
584	O157	O	0.610667646106	0.437383677324	0.782264538756
585	O158	O	0.737545204748	0.448053852428	0.743978058891
586	O159	O	0.868123072552	0.663364247906	0.738807844406
587	O160	O	0.804486946256	0.659923984523	0.572919825028
588	O161	O	0.693561580401	0.642446092186	0.463406778401
589	O162	O	0.585665077795	0.645832655882	0.578021174865
590	O163	O	0.610050752063	0.663227592380	0.776106615966
591	O164	O	0.737324331903	0.656149824821	0.739325609941
592	O165	O	0.807998122140	0.552307447997	0.687624771069
593	O166	O	0.572456425579	0.548735835334	0.703058012590
594	O167	O	0.915889956760	0.361263071301	0.886427924765
595	O168	O	0.909095811701	0.494076521274	0.922389466364
596	O169	O	0.902558890556	0.628148629373	0.922559956412
597	O170	O	0.690001782827	0.367511152981	0.884842404410
598	O171	O	0.689639076565	0.497311028094	0.911554765984
599	O172	O	0.693072267952	0.628894688283	0.921098888240
600	O173	O	0.493871103409	0.443983217972	0.707987657700
601	O174	O	0.491700284128	0.652482422985	0.713829794190
602	O175	O	0.131204631108	0.555867996107	0.249993726667
603	O176	O	0.191923641908	0.559239386864	0.417888997616
604	O177	O	0.299002537829	0.554730578691	0.532000672612
605	O178	O	0.401028742786	0.558929635265	0.410566092914
606	O179	O	0.389332340015	0.562616319235	0.217735469234
607	O180	O	0.262454789227	0.551946144537	0.256021944419
608	O181	O	0.131876934661	0.336635756822	0.261192163930
609	O182	O	0.195513064610	0.340076026711	0.427080149596

610	O183	O	0.306438410640	0.357553927415	0.536593201419
611	O184	O	0.414334932152	0.354167350891	0.421978859606
612	O185	O	0.389949236909	0.336772433375	0.223893448974
613	O186	O	0.262675657592	0.343850189799	0.260674370660
614	O187	O	0.192001843095	0.447692572480	0.312375217234
615	O188	O	0.427543579214	0.451264179430	0.296941996630
616	O189	O	0.084110039440	0.638736941665	0.113572066170
617	O190	O	0.090904196397	0.505923468403	0.077610529531
618	O191	O	0.097441099457	0.371851374747	0.077440043743
619	O192	O	0.309998248163	0.632488861099	0.115157567332
620	N1	N	0.484420833072	0.249987119270	0.938070350350
621	N2	N	0.515579156199	0.750012878477	0.061929641557
622	N3	N	0.015603494166	0.750218583273	0.436989529550
623	N4	N	0.984396511912	0.249781439778	0.563010464900
624	C1	C	0.504607900134	0.250136322818	0.830021853586
625	C2	C	0.577641162416	0.250660546779	0.809144636649
626	C3	C	0.588919282741	0.250402618174	0.697129278803
627	C4	C	0.408592720062	0.249846226208	0.948074530464
628	C5	C	0.367745000934	0.249598281807	0.853446906542
629	C6	C	0.294390826406	0.249634352548	0.878554336550
630	C7	C	0.513162035930	0.190731901968	0.993528607017
631	C8	C	0.501478245909	0.121859304247	0.952282927042
632	C9	C	0.541636807449	0.073576754954	0.015925114712
633	C10	C	0.495392093773	0.749863670038	0.169978139655
634	C11	C	0.422358833125	0.749339471010	0.190855366528
635	C12	C	0.411080715445	0.749597417502	0.302870725052
636	C13	C	0.591407269028	0.750153749830	0.051925455680
637	C14	C	0.632254989251	0.750401790373	0.146553077914
638	C15	C	0.705609162229	0.750365673816	0.121445640801
639	C16	C	0.486837960872	0.809268103275	0.006471394944
640	C17	C	0.498521765916	0.878140698179	0.047717074203
641	C18	C	0.458363204087	0.926423252349	0.984074894191
642	C19	C	0.995334113687	0.750426850967	0.328923257367

643	C20	C	0.922267864077	0.750665901044	0.308114400495
644	C21	C	0.910903358758	0.750714473728	0.196097208580
645	C22	C	0.091417718434	0.750171368636	0.446964983712
646	C23	C	0.132262612353	0.749622228069	0.352354754655
647	C24	C	0.205592466548	0.749488247401	0.377633634471
648	C25	C	0.987013910436	0.809412783538	0.492753701792
649	C26	C	0.998520826939	0.878295658142	0.451430691244
650	C27	C	0.958362864747	0.926575280580	0.515070332727
651	C28	C	0.004665891198	0.249573176753	0.671076736825
652	C29	C	0.077732140378	0.249334098719	0.691885596078
653	C30	C	0.089096641697	0.249285517677	0.803902789497
654	C31	C	0.908582288520	0.249828676908	0.553035007562
655	C32	C	0.867737392821	0.250377724887	0.647645236434
656	C33	C	0.794407539228	0.250511750762	0.622366353021
657	C34	C	0.012986086552	0.190587231300	0.507246303868
658	C35	C	0.001479157299	0.121704360885	0.548569320070
659	C36	C	0.041637120675	0.073424732471	0.484929690135
660	C37	C	0.487046306553	0.690756844874	0.006248188624
661	C38	C	0.498716758365	0.621906294745	0.047606323849
662	C39	C	0.458470850168	0.573543276974	0.984230418017
663	C40	C	0.512953670883	0.309243160963	0.993751796636
664	C41	C	0.501283216601	0.378093707572	0.952393650738
665	C42	C	0.541529120822	0.426456728586	0.015769558530
666	C43	C	0.013085402117	0.309099052430	0.507717402103
667	C44	C	0.001344474823	0.377892685837	0.549180069009
668	C45	C	0.041450584834	0.426272250779	0.485664748870
669	C46	C	0.986914616564	0.690900963160	0.492282587129
670	C47	C	0.998655547966	0.622107333289	0.450819910804
671	C48	C	0.958549432091	0.573727764687	0.514335216541
672	O_H1	O	0.310360894039	0.502688971847	0.088445263336
673	O_H2	O	0.306927746069	0.371105303048	0.078901095302
674	O_H3	O	0.506128898190	0.556016765086	0.292012330449
675	O_H4	O	0.508299704913	0.347517580585	0.286170251447

676	H1	H	0.565940287341	0.199751345615	0.997099337545
677	H2	H	0.518322508023	0.118540377205	0.875815844842
678	H3	H	0.543840322693	0.024559710927	0.981856526998
679	H4	H	0.592162180117	0.091021193556	0.023553919977
680	H5	H	0.494119836272	0.194848959229	0.068953810992
681	H6	H	0.449379560629	0.108535965276	0.952616660247
682	H7	H	0.521314143196	0.068345374582	0.090441804949
683	H8	H	0.396470088495	0.293623700671	0.991828513288
684	H9	H	0.378717209776	0.293265547116	0.808228196525
685	H10	H	0.265607343258	0.249578031211	0.810195953459
686	H11	H	0.280529135224	0.293516497275	0.921297192704
687	H12	H	0.481387252792	0.293741912095	0.797235358214
688	H13	H	0.601622957262	0.207337044155	0.841486567793
689	H14	H	0.641515257837	0.250639896761	0.680662340406
690	H15	H	0.568435914545	0.205878468339	0.662085822569
691	H16	H	0.469564304972	0.249284776744	0.604809725484
692	H17	H	0.434059707539	0.800248669262	0.002900670025
693	H18	H	0.481677511788	0.881459628122	0.124184160192
694	H19	H	0.456159699343	0.975440297990	0.018143477955
695	H20	H	0.407837827654	0.908978821178	0.976446104021
696	H21	H	0.505880155141	0.805151045296	0.931046188153
697	H22	H	0.550620453265	0.891464028604	0.047383328049
698	H23	H	0.478685859812	0.931654627671	0.909558197557
699	H24	H	0.603529895000	0.706376224119	0.008171581406
700	H25	H	0.621282772599	0.706734573771	0.191771888234
701	H26	H	0.734392650028	0.750422042097	0.189804018777
702	H27	H	0.719470835742	0.706483488723	0.078702863782
703	H28	H	0.518612728483	0.706258069552	0.202764621945
704	H29	H	0.398377051721	0.792662977723	0.158513426281
705	H30	H	0.358484740050	0.749360163987	0.319337665343
706	H31	H	0.431564103824	0.794121562574	0.337914167621
707	H32	H	0.530435716618	0.750715235935	0.395190233368
708	H33	H	0.934249122431	0.800362142035	0.496647929629

709	H34	H	0.981569512836	0.881508968652	0.375015032717
710	H35	H	0.955979157236	0.975526488187	0.480814591048
711	H36	H	0.907900278186	0.909016611969	0.522960055469
712	H37	H	0.006275899913	0.805267368482	0.568049409767
713	H38	H	0.050598292528	0.891697385866	0.451525267080
714	H39	H	0.978827947927	0.931984831012	0.589470810555
715	H40	H	0.103382790297	0.706697342169	0.491484704183
716	H41	H	0.121140573518	0.705729919682	0.307703009531
717	H42	H	0.234458981487	0.749232809752	0.309355084715
718	H43	H	0.219288281007	0.705737122088	0.420795569220
719	H44	H	0.018109817180	0.706623164163	0.296001655965
720	H45	H	0.898790332635	0.794368226326	0.340217568224
721	H46	H	0.858286030616	0.751038350945	0.179767500579
722	H47	H	0.931875782486	0.794855801711	0.160651171098
723	H48	H	0.031297314287	0.750382532123	0.104265127441
724	H49	H	0.065750876014	0.199637863555	0.503352077564
725	H50	H	0.018430463847	0.118491051770	0.624984982739
726	H51	H	0.044020818665	0.024473524878	0.519185432802
727	H52	H	0.092099710356	0.090983394774	0.477039979687
728	H53	H	0.993724100351	0.194732645896	0.431950594456
729	H54	H	0.949401689876	0.108302640563	0.548474733082
730	H55	H	0.021172045838	0.068015184445	0.410529206456
731	H56	H	0.896617222677	0.293302752716	0.508515389656
732	H57	H	0.878859441246	0.294269984750	0.692297082384
733	H58	H	0.765541022880	0.250767134768	0.690644901768
734	H59	H	0.780711739914	0.294262919926	0.579204505309
735	H60	H	0.981890204479	0.293376876034	0.703998327113
736	H61	H	0.101209655697	0.205631764119	0.659782429661
737	H62	H	0.141713969056	0.248961621716	0.820232503755
738	H63	H	0.068124200471	0.205144196308	0.839348822187
739	H64	H	0.968702682441	0.249617484235	0.895734860155
740	H65	H	0.434269626262	0.699738299099	0.002402241820
741	H66	H	0.481947838173	0.618708353231	0.124122530325

742	H67	H	0.456174397483	0.524616862511	0.018568575059
743	H68	H	0.407982151229	0.591054188275	0.976479603666
744	H69	H	0.506237807242	0.694907720457	0.930910599343
745	H70	H	0.550809327869	0.608562289375	0.047224804943
746	H71	H	0.478803054912	0.568073638225	0.909755734044
747	H72	H	0.603344571378	0.793829465839	0.007828525368
748	H73	H	0.621223234378	0.794279872589	0.191298247756
749	H74	H	0.719493006601	0.794150775639	0.078506885832
750	H75	H	0.517978894611	0.793841722731	0.202690555003
751	H76	H	0.399056352410	0.705436085747	0.159071062961
752	H77	H	0.432074085812	0.705542604953	0.338526304251
753	H78	H	0.565730351947	0.300261712784	0.997597752033
754	H79	H	0.518052142662	0.381291646467	0.875877446598
755	H80	H	0.543825579543	0.475383140915	0.981431395607
756	H81	H	0.592017818270	0.408945814765	0.023520388219
757	H82	H	0.493762167123	0.305092286331	0.069089383705
758	H83	H	0.449190645978	0.391437708680	0.952775161919
759	H84	H	0.521196909535	0.431926373058	0.090244237816
760	H85	H	0.396655419096	0.206170459763	0.992171351112
761	H86	H	0.378776766948	0.205720250383	0.808701632881
762	H87	H	0.280506994998	0.205849208642	0.921493007387
763	H88	H	0.482021114911	0.206158259460	0.797309444098
764	H89	H	0.600943627765	0.294563936591	0.840928953895
765	H90	H	0.567925895912	0.294457427158	0.661473712575
766	H91	H	0.065876451424	0.300141841707	0.504149698545
767	H92	H	0.018195581308	0.381095223321	0.625648716990
768	H93	H	0.043515621277	0.475290064197	0.519736504541
769	H94	H	0.092023384281	0.408975503882	0.478058266352
770	H95	H	0.994073108085	0.305091611236	0.432260565078
771	H96	H	0.949233089991	0.391164024148	0.548950364022
772	H97	H	0.021166325503	0.431438502597	0.411116195966
773	H98	H	0.896482312322	0.205896524462	0.509606970377
774	H99	H	0.878557437279	0.206722324474	0.692980195956

H100	H	0.780476364264	0.206604260197	0.579723213462
H101	H	0.981658339446	0.205805538643	0.703734771371
H102	H	0.101509881693	0.292865198743	0.659780096905
H103	H	0.068674119947	0.293704128715	0.839308374482
H104	H	0.934123566753	0.699858167472	0.495850300350
H105	H	0.981804447183	0.618904800211	0.374351259683
H106	H	0.956484399820	0.524709952721	0.480263455807
H107	H	0.907976631632	0.591024511798	0.521941689596
H108	H	0.005926914997	0.694908401943	0.567739422230
H109	H	0.050766932896	0.608835995700	0.451049622557
H110	H	0.978833684649	0.568561509563	0.588883772909
H111	H	0.103517692158	0.794103568918	0.490392915699
H112	H	0.121442558202	0.793277579151	0.307019694941
H113	H	0.219523629046	0.793395781529	0.420276684120
H114	H	0.018341647770	0.794194502994	0.296265234716
H115	H	0.898490140528	0.707134792474	0.340219903294
H116	H	0.931325893995	0.706295868275	0.160691626012

Structural Data for DFT optimized Silicalite-1 (without TPA^+OH^-)

data_S1-DFT
_cell_length_a 20.223082
_cell_length_b 19.867162
_cell_length_c 13.345321
_cell_angle_alpha 90
_cell_angle_beta 90
_cell_angle_gamma 90
_cell_volume 5361.8196
_symmetry_space_group_name_H-M P1
loop_
    _symmetry_equiv_pos_as_xyz
    'x, y, z '
loop_

_atom_site_label
_atom_site_type_symbol
_atom_site_fract_x
_atom_site_fract_y
_atom_site_fract_z
T1(a) Si 0.423253935068 0.071114668310 0.688755454200
T1(b) Si 0.076775726306 0.928430701199 0.189216467569
T1(c) Si 0.923224273545 0.428430702709 0.810783532326
T1(d) Si 0.576746065174 0.571114666851 0.311244545163
T1(e) Si 0.576746065090 0.928885334000 0.311244541109
T1(f) Si 0.923224278243 0.071569300703 0.810783535353
T1(g) Si 0.076775722212 0.571569298911 0.189216465876
T1(h) Si 0.423253934776 0.428885335762 0.688755458906
T2(a) Si 0.325323836431 0.033717925902 0.847976718155
T2(b) Si 0.174388115165 0.966179746513 0.348815209548
T2(c) Si 0.825611885088 0.466179747606 0.651184789935
T2(d) Si 0.674676165191 0.533717923969 0.152023284618
T2(e) Si 0.674676170551 0.966282072981 0.152023291856
T2(f) Si 0.825611886413 0.033820256673 0.651184788781
T2(g) Si 0.174388115249 0.533820256044 0.348815209773
T2(h) Si 0.325323829538 0.466282072512 0.847976707881
T3(a) Si 0.277864853962 0.054932472580 0.063974523902
T3(b) Si 0.221899311010 0.945089107055 0.564056684864
T3(c) Si 0.778100687185 0.445089106386 0.435943314102
T3(d) Si 0.722135148302 0.554932467400 0.936025478540
T3(e) Si 0.722135152713 0.945067545707 0.936025478143
T3(f) Si 0.778100690335 0.054910892099 0.435943314410
T3(g) Si 0.221899311376 0.554910892527 0.564056685890
T3(h) Si 0.277864846565 0.445067546140 0.063974520898
T4(a) Si 0.123315371960 0.051587529170 0.048615456676
T4(b) Si 0.376545568440 0.947835022118 0.547318647030
T4(c) Si 0.623454430309 0.447835018983 0.452681351456
T4(d) Si 0.876684631062 0.551587531808 0.951384542552

840	T4(e)	Si	0.876684639008	0.948412468731	0.951384544193
841	T4(f)	Si	0.623454434744	0.052164975320	0.452681352318
842	T4(g)	Si	0.376545566907	0.552164978717	0.547318647809
843	T4(h)	Si	0.123315360586	0.448412466189	0.048615456429
844	T5(a)	Si	0.072639489644	0.036516417342	0.824758106778
845	T5(b)	Si	0.427492863991	0.963432470722	0.323744624352
846	T5(c)	Si	0.572507135891	0.463432470259	0.676255374322
847	T5(d)	Si	0.927360510910	0.536516418757	0.175241892900
848	T5(e)	Si	0.927360513971	0.963483581671	0.175241898445
849	T5(f)	Si	0.572507136781	0.036567528608	0.676255374599
850	T5(g)	Si	0.427492863333	0.536567528900	0.323744624929
851	T5(h)	Si	0.072639485950	0.463483579904	0.824758101862
852	T6(a)	Si	0.202511776390	0.067628411350	0.716932987262
853	T6(b)	Si	0.297400991461	0.932444200269	0.216631625729
854	T6(c)	Si	0.702599008583	0.432444197521	0.783368371461
855	T6(d)	Si	0.797488224871	0.567628411914	0.283067014484
856	T6(e)	Si	0.797488229445	0.932371585891	0.283067022704
857	T6(f)	Si	0.702599006724	0.067555820831	0.783368373028
858	T6(g)	Si	0.297400993108	0.567555821944	0.216631623571
859	T6(h)	Si	0.202511770713	0.432371586133	0.716932977258
860	T7(a)	Si	0.419761572791	0.827626064276	0.682386453507
861	T7(b)	Si	0.079847002198	0.172577320460	0.181702212318
862	T7(c)	Si	0.920152998930	0.672577322654	0.818297787314
863	T7(d)	Si	0.580238425829	0.327626061750	0.317613547287
864	T7(e)	Si	0.580238434518	0.172373930625	0.317613538168
865	T7(f)	Si	0.920152999721	0.827422684694	0.818297780675
866	T7(g)	Si	0.079846999795	0.327422682651	0.181702219377
867	T7(h)	Si	0.419761566570	0.672373933343	0.682386461450
868	T8(a)	Si	0.315015242598	0.875963866651	0.836872162153
869	T8(b)	Si	0.184791158969	0.124222634597	0.336593915411
870	T8(c)	Si	0.815208841452	0.624222635664	0.663406084717
871	T8(d)	Si	0.684984756305	0.375963863344	0.163127833606
872	T8(e)	Si	0.684984764241	0.124036132654	0.163127825048

T8(f)	Si	0.815208842638	0.875777366818	0.663406088321
T8(g)	Si	0.184791157105	0.375777365615	0.336593912039
T8(h)	Si	0.315015237074	0.624036132312	0.836872173491
T9(a)	Si	0.272830531632	0.827832658917	0.042111503320
T9(b)	Si	0.227368749986	0.172649909947	0.541899219442
T9(c)	Si	0.772631251241	0.672649911054	0.458100780768
T9(d)	Si	0.727169468254	0.327832655243	0.957888490813
T9(e)	Si	0.727169467933	0.172167356485	0.957888484998
T9(f)	Si	0.772631254450	0.827350088377	0.458100787557
T9(g)	Si	0.227368746421	0.327350087954	0.541899212248
T9(h)	Si	0.272830532789	0.672167358045	0.042111511825
T10(a)	Si	0.117850363540	0.828091797344	0.020377331411
T10(b)	Si	0.383105410412	0.172598986554	0.521932913865
T10(c)	Si	0.616894589465	0.672598986921	0.478067084981
T10(d)	Si	0.882149636000	0.328091798728	0.979622669556
T10(e)	Si	0.882149627391	0.171908192192	0.979622663411
T10(f)	Si	0.616894589692	0.827401018701	0.478067082380
T10(g)	Si	0.383105410476	0.327401019018	0.521932918721
T10(h)	Si	0.117850373365	0.671908190536	0.020377336993
T11(a)	Si	0.066572125826	0.879233300997	0.811088192371
T11(b)	Si	0.433821996998	0.121048108718	0.312434022269
T11(c)	Si	0.566178003279	0.621048109030	0.687565976982
T11(d)	Si	0.933427873640	0.379233301541	0.188911808318
T11(e)	Si	0.933427873496	0.120766697090	0.188911802781
T11(f)	Si	0.566178004708	0.878951890330	0.687565977416
T11(g)	Si	0.433821994462	0.378951890320	0.312434023715
T11(h)	Si	0.066572126983	0.620766696250	0.811088197376
T12(a)	Si	0.195029973033	0.828692481332	0.707836504660
T12(b)	Si	0.305039823490	0.171223153936	0.209485719864
T12(c)	Si	0.694960176644	0.671223152456	0.790514281110
T12(d)	Si	0.804970026389	0.328692480481	0.292163494846
T12(e)	Si	0.804970029756	0.171307517953	0.292163499731
T12(f)	Si	0.694960180778	0.828776855064	0.790514274471

T12(g)	Si	0.305039819232	0.328776855904	0.209485726173
T12(h)	Si	0.195029970783	0.671307518663	0.707836500448
O1	O	0.502877307312	0.072843651152	0.703672964802
O2	O	0.996990951676	0.926908910523	0.201711683773
O3	O	0.003009048052	0.426908909214	0.798288316797
O4	O	0.497122692985	0.572843651041	0.296327035108
O5	O	0.497122692901	0.927156348743	0.296327035580
O6	O	0.003009052779	0.073091091521	0.798288318970
O7	O	0.996990947844	0.573091091893	0.201711681847
O8	O	0.502877306763	0.427156348959	0.703672963925
O9	O	0.388753023229	0.072537380820	0.799393578169
O10	O	0.110880380958	0.927146647332	0.300493433564
O11	O	0.889119619018	0.427146647860	0.699506566211
O12	O	0.611246977261	0.572537378605	0.200606421764
O13	O	0.611246978833	0.927462621783	0.200606421022
O14	O	0.889119621708	0.072853352890	0.699506569021
O15	O	0.110880378708	0.572853352104	0.300493432237
O16	O	0.388753021973	0.427462622412	0.799393579712
O17	O	0.399648682679	0.138618996437	0.629564681014
O18	O	0.100355326597	0.861558222181	0.128077684119
O19	O	0.899644672360	0.361558225412	0.871922317837
O20	O	0.600351317574	0.638618994570	0.370435319076
O21	O	0.600351318103	0.861381005682	0.370435313830
O22	O	0.899644670333	0.138441776903	0.871922312434
O23	O	0.100355330018	0.638441774054	0.128077686839
O24	O	0.399648681418	0.361381007614	0.629564686192
O25	O	0.399743115570	0.003367719541	0.629631612675
O26	O	0.100206011977	0.996810533986	0.131924064187
O27	O	0.899793988869	0.496810536271	0.868075933857
O28	O	0.600256885024	0.503367717865	0.370368386261
O29	O	0.600256885088	0.996632280378	0.370368385714
O30	O	0.899793993378	0.003189465239	0.868075934801
O31	O	0.100206006448	0.503189463180	0.131924066450

939	O32	O	0.399743115703	0.496632282719	0.629631614721
940	O33	O	0.326901903919	0.041744333151	0.969195472585
941	O34	O	0.172018000080	0.958452735514	0.470284666828
942	O35	O	0.827981995336	0.458452738454	0.529715333044
943	O36	O	0.673098097669	0.541744332739	0.030804530299
944	O37	O	0.673098104517	0.958255668535	0.030804537515
945	O38	O	0.827982004766	0.041547264959	0.529715329920
946	O39	O	0.172018000827	0.541547262452	0.470284668379
947	O40	O	0.326901894924	0.458255668998	0.969195462132
948	O41	O	0.329997866181	0.954856764580	0.814776553940
949	O42	O	0.169784210859	0.045189084259	0.316626025886
950	O43	O	0.830215789102	0.545189085517	0.683373975028
951	O44	O	0.670002134655	0.454856761690	0.185223444060
952	O45	O	0.670002134932	0.045143236422	0.185223445258
953	O46	O	0.830215788716	0.954810917412	0.683373972361
954	O47	O	0.169784212031	0.454810916134	0.316626027759
955	O48	O	0.329997865029	0.545143236291	0.814776553603
956	O49	O	0.256406598524	0.066121403263	0.807562163353
957	O50	O	0.243213081557	0.934109072675	0.306913485970
958	O51	O	0.756786918433	0.434109074572	0.693086512501
959	O52	O	0.743593402138	0.566121402086	0.192437840214
960	O53	O	0.743593403874	0.933878594980	0.192437853462
961	O54	O	0.756786918997	0.065890929832	0.693086513910
962	O55	O	0.243213081463	0.565890929439	0.306913483407
963	O56	O	0.256406596151	0.433878595146	0.807562146486
964	O57	O	0.288283970955	0.131519053602	0.105385511858
965	O58	O	0.211577519445	0.868174464893	0.603590681453
966	O59	O	0.788422481173	0.368174465170	0.396409317408
967	O60	O	0.711716029416	0.631519047164	0.894614484657
968	O61	O	0.711716029846	0.868480966939	0.894614473005
969	O62	O	0.788422479709	0.131825535811	0.396409322541
970	O63	O	0.211577519846	0.631825535258	0.603590678388
971	O64	O	0.288283969797	0.368480968339	0.105385528419

972	O65	O	0.201997989055	0.042247084334	0.026533513431
973	O66	O	0.297710350050	0.955583761219	0.524542638382
974	O67	O	0.702289648249	0.455583754127	0.475457361333
975	O68	O	0.798002014189	0.542247083383	0.973466487153
976	O69	O	0.798002020864	0.957752916476	0.973466491042
977	O70	O	0.702289652814	0.044416237140	0.475457364045
978	O71	O	0.297710349120	0.544416243971	0.524542636254
979	O72	O	0.201997978394	0.457752915917	0.026533509220
980	O73	O	0.295426529913	0.002868451065	0.154139403406
981	O74	O	0.204885263230	0.996609541967	0.655566127911
982	O75	O	0.795114736423	0.496609541876	0.344433872658
983	O76	O	0.704573469603	0.502868446163	0.845860599989
984	O77	O	0.704573470176	0.997131571515	0.845860604372
985	O78	O	0.795114738752	0.003390458969	0.344433871549
986	O79	O	0.204885261871	0.503390459488	0.655566128428
987	O80	O	0.295426528825	0.497131572149	0.154139393312
988	O81	O	0.107895880213	0.127439445438	0.088848720731
989	O82	O	0.392390831694	0.872123116170	0.587996460183
990	O83	O	0.607609167431	0.372123112471	0.412003540949
991	O84	O	0.892104120415	0.627439449011	0.911151280370
992	O85	O	0.892104124816	0.872560552992	0.911151279239
993	O86	O	0.607609170991	0.127876881263	0.412003538858
994	O87	O	0.392390829954	0.627876884882	0.587996460325
995	O88	O	0.107895875787	0.372560549876	0.088848720297
996	O89	O	0.082870576740	0.037907487592	0.945850405824
997	O90	O	0.416972790537	0.961672602086	0.444580428174
998	O91	O	0.583027209216	0.461672600827	0.555419570665
999	O92	O	0.917129425203	0.537907489932	0.054149593831
1000	O93	O	0.917129428511	0.962092514296	0.054149598455
1001	O94	O	0.583027208963	0.038327397033	0.555419569466
1002	O95	O	0.416972791244	0.538327399268	0.444580429912
1003	O96	O	0.082870571034	0.462092511558	0.945850402040
1004	O97	O	0.129853386155	0.079034336172	0.766792901602

1005	O98	O	0.370560197313	0.921271235051	0.264767651719
1006	O99	O	0.629439802539	0.421271233259	0.735232345779
1007	O100	O	0.870146614824	0.579034336912	0.233207099387
1008	O101	O	0.870146617800	0.920965660319	0.233207101350
1009	O102	O	0.629439798879	0.078728776460	0.735232345539
1010	O103	O	0.370560200409	0.578728773687	0.264767653030
1011	O104	O	0.129853382833	0.420965659403	0.766792899159
1012	O105	O	0.073046258785	0.958959218800	0.784051925210
1013	O106	O	0.427047207518	0.041417050070	0.285692580965
1014	O107	O	0.572952792196	0.541417049894	0.714307420031
1015	O108	O	0.926953741294	0.458959219767	0.215948073681
1016	O109	O	0.926953741838	0.041040780122	0.215948080515
1017	O110	O	0.572952794465	0.958582949422	0.714307420279
1018	O111	O	0.427047206054	0.458582949397	0.285692578950
1019	O112	O	0.073046257964	0.541040778804	0.784051918489
1020	O113	O	0.218892868950	0.131638197454	0.645793024349
1021	O114	O	0.281027097240	0.867624754849	0.147207435790
1022	O115	O	0.718972904353	0.367624751794	0.852792558770
1023	O116	O	0.781107131381	0.631638197907	0.354206975996
1024	O117	O	0.781107130763	0.868361804932	0.354206985085
1025	O118	O	0.718972905312	0.132375264831	0.852792552888
1026	O119	O	0.281027096102	0.632375266946	0.147207445471
1027	O120	O	0.218892869460	0.368361806795	0.645793012645
1028	O121	O	0.492279584111	0.854085119712	0.718798233238
1029	O122	O	0.007580101178	0.145741916908	0.217631097798
1030	O123	O	0.992419899717	0.645741918287	0.782368901752
1031	O124	O	0.507720414114	0.354085116611	0.281201769744
1032	O125	O	0.507720418549	0.145914878220	0.281201764334
1033	O126	O	0.992419898911	0.854258085357	0.782368893412
1034	O127	O	0.007580100234	0.354258083485	0.217631105164
1035	O128	O	0.492279582400	0.645914880651	0.718798235119
1036	O129	O	0.369010895219	0.830340571467	0.777464511809
1037	O130	O	0.131448623405	0.169913207739	0.275720923513

1038	O131	O	0.868551377278	0.669913208102	0.724279076944
1039	O132	O	0.630989101305	0.330340572383	0.222535486700
1040	O133	O	0.630989116095	0.169659431176	0.222535484841
1041	O134	O	0.868551377935	0.830086792708	0.724279075633
1042	O135	O	0.131448621501	0.330086792497	0.275720924248
1043	O136	O	0.369010886160	0.669659430501	0.777464515541
1044	O137	O	0.424071822074	0.749999998772	0.644298719454
1045	O138	O	0.073483457615	0.250000002300	0.142767964030
1046	O139	O	0.926516543003	0.750000004585	0.857232035902
1047	O140	O	0.575928178390	0.249999995857	0.355701279992
1048	O141	O	0.320064874271	0.861502276999	0.956792045249
1049	O142	O	0.180238112039	0.139094206847	0.456202882808
1050	O143	O	0.819761888628	0.639094206525	0.543797116750
1051	O144	O	0.679935125665	0.361502274447	0.043207949835
1052	O145	O	0.679935129433	0.138497730521	0.043207943211
1053	O146	O	0.819761894918	0.860905788466	0.543797124595
1054	O147	O	0.180238104509	0.360905785950	0.456202875143
1055	O148	O	0.320064870147	0.638497727924	0.956792055860
1056	O149	O	0.240391678899	0.857900293817	0.798921198711
1057	O150	O	0.259832275990	0.141972184266	0.300665997791
1058	O151	O	0.740167724539	0.641972187467	0.699334000051
1059	O152	O	0.759608319963	0.357900291884	0.201078800697
1060	O153	O	0.759608328434	0.142099704135	0.201078797115
1061	O154	O	0.740167722576	0.858027813605	0.699333984443
1062	O155	O	0.259832277143	0.358027811421	0.300666017228
1063	O156	O	0.240391673371	0.642099706365	0.798921202435
1064	O157	O	0.294751823970	0.750000007132	0.059501875069
1065	O158	O	0.204745551390	0.249999999079	0.561357019348
1066	O159	O	0.795254450731	0.750000000111	0.438642983410
1067	O160	O	0.705248175422	0.250000004258	0.940498112732
1068	O161	O	0.197140164506	0.832822751408	0.000916482376
1069	O162	O	0.303686534006	0.170163074167	0.502820204632
1070	O163	O	0.696313466355	0.670163078954	0.497179792258

1071	O164	O	0.802859834386	0.332822752424	0.999083517392
1072	O165	O	0.802859830158	0.167177250616	0.999083511397
1073	O166	O	0.696313467354	0.829836923422	0.497179796327
1074	O167	O	0.303686533131	0.329836925214	0.502820201889
1075	O168	O	0.197140170207	0.667177248492	0.000916488056
1076	O169	O	0.093613310118	0.749999993748	0.020966038044
1077	O170	O	0.409579238372	0.250000001626	0.523337526523
1078	O171	O	0.590420761104	0.750000001943	0.476662474646
1079	O172	O	0.906386691148	0.249999994921	0.979033961776
1080	O173	O	0.079815563167	0.868307332889	0.930916026928
1081	O174	O	0.420460459786	0.131700887938	0.432407218567
1082	O175	O	0.579539540323	0.631700886805	0.567592779829
1083	O176	O	0.920184436769	0.368307334606	0.069083973349
1084	O177	O	0.920184434633	0.131692665193	0.069083970142
1085	O178	O	0.579539537816	0.868299117725	0.567592778720
1086	O179	O	0.420460461175	0.368299119049	0.432407222404
1087	O180	O	0.079815565847	0.631692663466	0.930916030165
1088	O181	O	0.117840060130	0.836746678182	0.741081302720
1089	O182	O	0.382086587059	0.163427320998	0.243242999033
1090	O183	O	0.617913412960	0.663427322322	0.756756999715
1091	O184	O	0.882159939489	0.336746678066	0.258918697145
1092	O185	O	0.882159941917	0.163253321949	0.258918696973
1093	O186	O	0.617913415824	0.836572679273	0.756756996651
1094	O187	O	0.382086584350	0.336572678861	0.243243003147
1095	O188	O	0.117840058553	0.663253321863	0.741081303852
1096	O189	O	0.211737370713	0.750000000362	0.687608660256
1097	O190	O	0.288249080338	0.250000003971	0.189868239123
1098	O191	O	0.711750919439	0.750000003398	0.810131762923
1099	O192	O	0.788262629208	0.249999999144	0.312391339579

SEM images

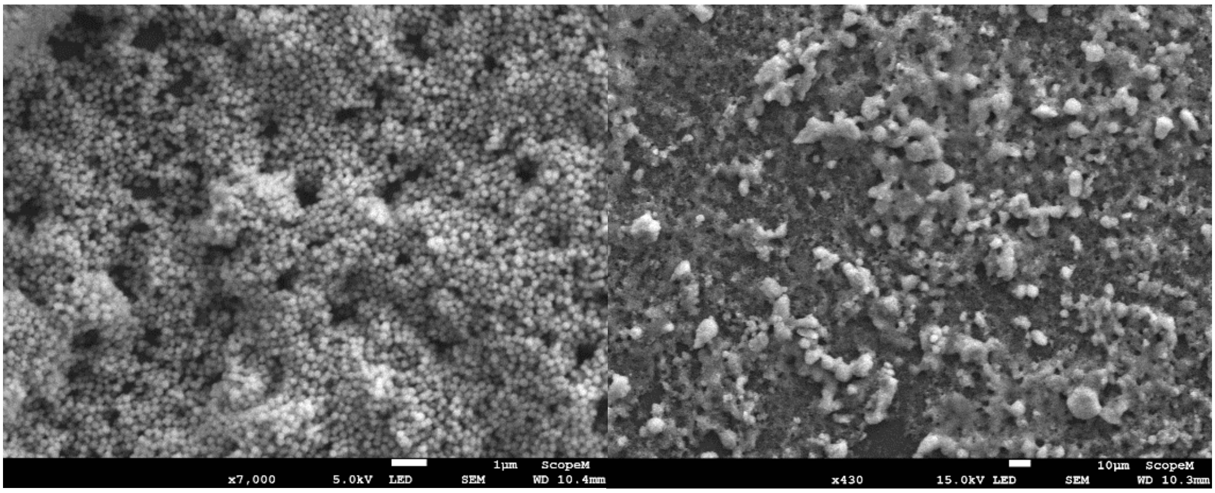

Supplementary Figure 7. SEM image of sample TS-1A (left) and TS-1B (right), as prepared for the AXRD measurement.

Structure of possible Ti-peroxo species

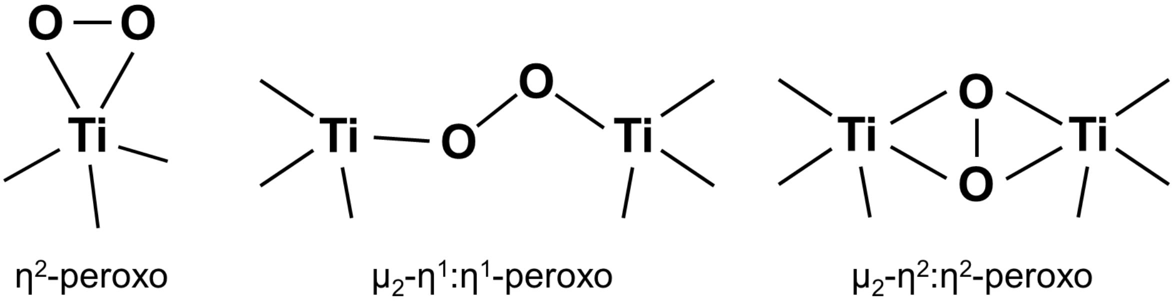

Supplementary Figure 8. Structure of possible monomeric and dimeric Ti-peroxo species.

Supplementary References

1. Signorile, M. *et al.* Effect of Ti Speciation on Catalytic Performance of TS-1 in the Hydrogen Peroxide to Propylene Oxide Reaction. *J. Phys. Chem. C* **122**, 9021–9034 (2018).
2. Su, J. *et al.* Amorphous Ti species in titanium silicalite-1: Structural features, chemical properties, and inactivation with sulfosalt. *J. Catal.* **288**, 1–7 (2012).
3. Guo, Q., Feng, Z., Li, G., Fan, F. & Li, C. Finding the 'missing components' during the synthesis of TS-1 zeolite by UV resonance raman spectroscopy. *J. Phys. Chem. C* **117**, 2844–2848 (2013).

4. Zuo, Y. *et al.* Role of pentahedrally coordinated titanium in titanium silicalite-1 in propene epoxidation. *RSC Adv.* **5**, 17897–17904 (2015).

5. Signorile, M. *et al.* Titanium Defective Sites in TS-1: Structural Insights by Combining Spectroscopy and Simulation. *Angew. Chemie - Int. Ed.* **59**, 18145–18150 (2020).

6. Ricchiardi, G. *et al.* Vibrational structure of titanium silicate catalysts. A spectroscopic and theoretical study. *J. Am. Chem. Soc.* **123**, 11409–11419 (2001).

7. Ohsaka, T., Izumi, F. & Fujiki, Y. Raman spectrum of anatase, TiO2. *J. Raman Spectrosc.* **7**, 321–324 (1978).

8. Robson, H., Lillerud, K. P. & Patterns, X. *Verified Syntheses of Zeolitic Materials*. *Verified Syntheses of Zeolitic Materials* (Elsevier, 2001).

9. Bordiga, S. *et al.* Hydroxyls nests in defective silicalites and strained structures derived upon dehydroxylation: Vibrational properties and theoretical modelling. *Top. Catal.* **15**, 43–52 (2001).

10. Li, W. *et al.* Differential pair distribution function study of the structure of arsenate adsorbed on nanocrystalline γ-alumina. *Environ. Sci. Technol.* **45**, 9687–9692 (2011).

1. Signorile, M. *et al.* Effect of Ti Speciation on Catalytic Performance of TS-1 in the Hydrogen Peroxide to Propylene Oxide Reaction. *J. Phys. Chem. C* **122**, 9021–9034 (2018).

2. Su, J. *et al.* Amorphous Ti species in titanium silicalite-1: Structural features, chemical properties, and inactivation with sulfosalt. *J. Catal.* **288**, 1–7 (2012).

3. Guo, Q., Feng, Z., Li, G., Fan, F. & Li, C. Finding the 'missing components' during the synthesis of TS-1 zeolite by UV resonance raman spectroscopy. *J. Phys. Chem. C* **117**, 2844–2848 (2013).

4. Zuo, Y. *et al.* Role of pentahedrally coordinated titanium in titanium silicalite-1 in propene epoxidation. *RSC Adv.* **5**, 17897–17904 (2015).

5. Signorile, M. *et al.* Titanium Defective Sites in TS-1: Structural Insights by

Combining Spectroscopy and Simulation. *Angew. Chemie - Int. Ed.* **59**, 18145–18150 (2020).

6. Ricchiardi, G. *et al.* Vibrational structure of titanium silicate catalysts. A spectroscopic and theoretical study. *J. Am. Chem. Soc.* **123**, 11409–11419 (2001).

7. Ohsaka, T., Izumi, F. & Fujiki, Y. Raman spectrum of anatase, TiO2. *J. Raman Spectrosc.* **7**, 321–324 (1978).

8. Robson, H., Lillerud, K. P. & Patterns, X. *Verified Syntheses of Zeolitic Materials*. *Verified Syntheses of Zeolitic Materials* (Elsevier, 2001).

9. Bordiga, S. *et al.* Hydroxyls nests in defective silicalites and strained structures derived upon dehydroxylation: Vibrational properties and theoretical modelling. *Top. Catal.* **15**, 43–52 (2001).

10. Li, W. *et al.* Differential pair distribution function study of the structure of arsenate adsorbed on nanocrystalline γ-alumina. *Environ. Sci. Technol.* **45**, 9687–9692 (2011).
